# Gene Resistance to Transcriptional Reprogramming following Nuclear Transfer Is Directly Mediated by Multiple Chromatin-Repressive Pathways

**DOI:** 10.1016/j.molcel.2017.01.030

**Published:** 2017-03-02

**Authors:** Jerome Jullien, Munender Vodnala, Vincent Pasque, Mami Oikawa, Kei Miyamoto, George Allen, Sarah Anne David, Vincent Brochard, Stan Wang, Charles Bradshaw, Haruhiko Koseki, Vittorio Sartorelli, Nathalie Beaujean, John Gurdon

**Affiliations:** 1 Wellcome Trust/Cancer Research UK Gurdon Institute, University of Cambridge, Cambridge CB2 1QN, UK; 2 Department of Development and Regeneration, KU Leuven, University of Leuven, Herestraat 49, 3000 Leuven, Belgium; 3 Laboratory of Molecular Developmental Biology, Graduate School of Biology-Oriented Science and Technology, Kinki University, Wakayama 649-6493, Japan; 4 UMR BDR, INRA, ENVA, Université Paris Saclay, 78350 Jouy en Josas, France; 5 Laboratory of Muscle Stem Cells and Gene Regulation, National Institute of Arthritis, Musculoskeletal and Skin Diseases (NIAMS), NIH, Bethesda, MD 20892, USA; 6 RIKEN Center for Integrative Medical Sciences, Laboratory for Developmental Genetics, North Research Building, 1-7-22 Suehiro-cho, Tsurumi-ku, Yokohama City, Kanagawa 230-0045, Japan

**Keywords:** transcriptional reprogramming, oocyte, xenopus, nuclear transfer, resistance, epigenetic, chromatin

## Abstract

Understanding the mechanism of resistance of genes to reactivation will help improve the success of nuclear reprogramming. Using mouse embryonic fibroblast nuclei with normal or reduced DNA methylation in combination with chromatin modifiers able to erase H3K9me3, H3K27me3, and H2AK119ub1 from transplanted nuclei, we reveal the basis for resistance of genes to transcriptional reprogramming by oocyte factors. A majority of genes is affected by more than one type of treatment, suggesting that resistance can require repression through multiple epigenetic mechanisms. We classify resistant genes according to their sensitivity to 11 chromatin modifier combinations, revealing the existence of synergistic as well as adverse effects of chromatin modifiers on removal of resistance. We further demonstrate that the chromatin modifier USP21 reduces resistance through its H2AK119 deubiquitylation activity. Finally, we provide evidence that H2A ubiquitylation also contributes to resistance to transcriptional reprogramming in mouse nuclear transfer embryos.

## Highlights

• Identification of genes resistant to direct transcriptional reprogramming
• Determination of resistant gene sensitivity to 11 chromatin modifier combinations
• USP21 removes resistance through its H2AK119 deubiquitylation activity
• USP21 improves the reprogramming of gene expression in two-cell-stage mouse embryos

## Graphical Abstract

**Figure:**
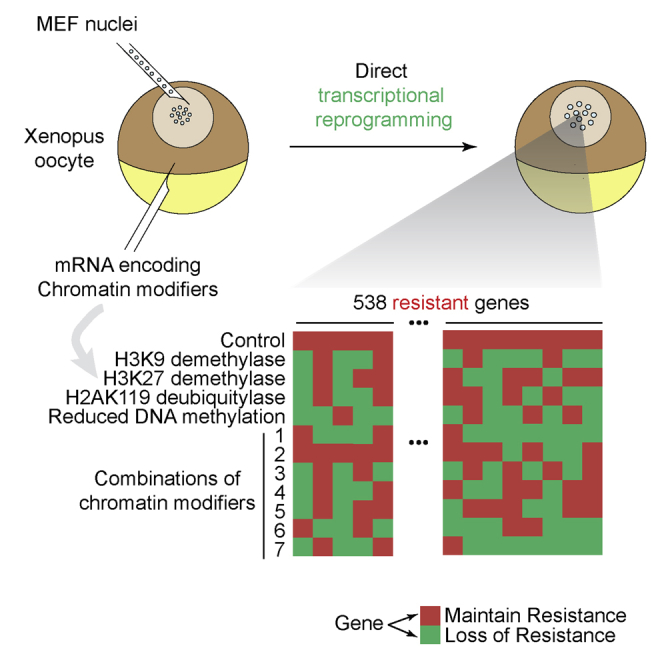

## Teaser

Using nuclear transfer to *Xenopus* oocytes, Jullien et al. identify genes in MEF nuclei whose epigenetic configuration directly resists transcriptional reprogramming by oocyte factors. They show that loss of resistance can be achieved by a combinatorial action of chromatin modifiers, which alter H2A ubiquitylation, H3K9, H3K27, and DNA methylation levels.

## Introduction

Complete nuclear reprogramming is an inefficient process, irrespective of the procedure used (Gurdon, 1960, Pereira et al., 2008, Takahashi and Yamanaka, 2006). The resistance to reprogramming is thought to come from the cellular mechanisms that ensure the stability of the differentiated state. Understanding how the differentiated state of a cell resists reprogramming is key to our ability to improve the reprogramming procedure. Importantly, disruption of cell identity can account for a large part in the development of many diseases and in aging (Roy and Hebrok, 2015, Sun et al., 2014). Therefore, analysis of resistance to nuclear reprogramming provides a unique opportunity to identify the stabilization mechanisms that are perturbed in these situations.

Previous work has indicated that resistance to reprogramming is partly linked to failure in gene activation. Numerous studies have shown that the epigenetic configuration of somatic nuclei can constitute a barrier to reprogramming (Cheloufi et al., 2015, Mansour et al., 2012, Matoba et al., 2014, Mikkelsen et al., 2008, Onder et al., 2012, Soufi et al., 2012). However, because of the multiple cellular processes occurring at once in reprogramming, a clear picture is lacking on (1) how directly the epigenetic configuration of a gene will affect its reactivation and (2) how multiple epigenetic marks regulate reactivation at a single-gene level. We have recently described a nuclear transfer procedure in which mouse nuclei are transcriptionally reprogrammed toward an oocyte type of transcription (Jullien et al., 2014). Importantly, this transcriptional reprogramming is directly mediated by *Xenopus* oocyte factors and in the absence of cell division. Here, using this direct assay, we first identify genes that are resistant to transcriptional reprogramming. Increased expression of these genes upon interference with the chromatin status of transplanted nuclei reveals how resistance is achieved on a single-gene basis. As a result, classes of resistant genes are identified. At a single-gene level, we describe cases of neutral, adverse, or synergistic effect of a combination of chromatin modifier expression on transcription. We further demonstrate that, in the case of USP21, the effect on resistance removal can be attributed to the deubiquitylation of H2AK119 from transplanted chromatin. Finally, we provide evidence that resistance to transcriptional reprogramming following nuclear transfer to egg can also be alleviated by USP21 expression.

## Results

### Identification of Genes Resistant to Transcriptional Reprogramming

**Figure 1:**
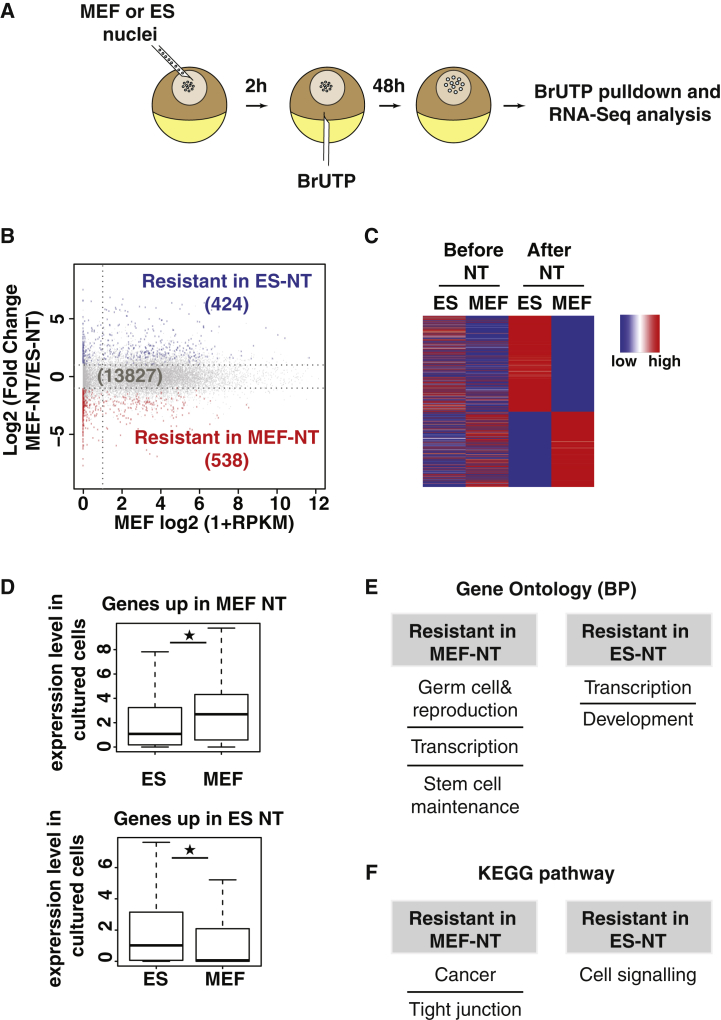
Identification of Genes Resisting Transcriptional Reprogramming by Oocytes (A) Schematic representation shows the nuclear transplantation strategy used to identify genes resistant to reactivation by *Xenopus* oocytes. (B) MA plot shows log fold change (logFC, y axis) in gene expression between MEF-NT versus ES-NT against the expression in cultured MEFs (x axis, log2 reads per kilobase per million mapped reads [RPKM]; data from Reddington et al., 2013). Red and blue dots, genes differentially expressed (FDR < 0.05, two experiments, n = 32 NT samples per condition). (C) Heatmap representing expression of the differentially expressed genes (rows, *Z* score of normalized expression level) between MEF-NT and ES-NT in ES and MEF before and 48 hr after NT (columns, genes sorted according to fold change between MEF-NT to ES-NT). Expression data for cells before NT are from Reddington et al. (2013) and Bulut-Karslioglu et al. (2014). (D) Boxplot shows the gene expression level before and after NT of genes resistant in MEF-NT (538 genes, top) or resistant in ES-NT (424 genes, bottom). ★p value < 10^−6^, Wicoxon rank-sum test. (E and F) The top (E) gene ontology terms and (F) KEGG pathways enriched in the list of differentially expressed (DE) genes restricted in MEF-NT and ES-NT. See also Figure S1 and Tables S1 and S2.

Mammalian nuclei transplanted to *Xenopus* oocyte are reprogrammed from a somatic to an oocyte type of transcription (Jullien et al., 2014). In the context of such a genome-wide change in transcription, the proportion of genes that resist the reprogramming activity of the oocyte is unknown. To identify such genes, we have compared the reprogramming of mouse embryonic stem cell nuclei to that of fibroblast nuclei transplanted to oocytes (Figure 1A). In this experimental setup, we can compare the transcription from two types of transplanted chromatin exposed to the same oocyte factors. To label RNAs produced after transplantation, we injected BrUTP to the oocyte 2 hr after nuclear transfer (Jullien et al., 2014). At 48 hr after nuclear transfer, labeled RNAs were immunopurified and subjected to RNA sequencing (RNA-seq) analysis. Such procedure excludes somatic cell transcripts carried over during nuclear transplantation, and, therefore, it focuses our analysis exclusively on oocyte factor-mediated transcription. Hierarchical clustering of gene expression confirmed that the BrUTP injection allowed the purification of labeled RNAs, as the samples injected with BrUTP clustered away from the control samples generated without BrUTP (Figure S1A).

Transplanted embryonic stem cell (ESC) nuclei share a large number of transcribed genes with fibroblasts (gray dots, 13,827 genes; Figure 1B; Table S1). This indicates that the oocyte has an efficient reprogramming activity, since nuclei originating from cells as different as an embryonic stem cell and an embryonic fibroblast express a large number of genes after nuclear transfer (of all genes expressed after nuclear transfer, 93% are similarly expressed between ESC and mouse embryonic fibroblast (MEF) nuclei, as compared to 52% before transplantation). However, we found that a number of genes are differentially expressed (false discovery rate [FDR] < 0.05) between transplanted ESC and MEF nuclei (Figure 1B, blue and red dots; Table S1). The qRT-PCR analysis confirmed differential expression of 15/17 genes tested, indicating the robustness of our RNA-seq assay (Figure S1B).

The oocyte better transcribes these genes from one type of transplanted nucleus than from the other. This suggests that differences in the epigenetic constitution between these two types of nuclei is such that some genes are resistant to the oocyte reprogramming factors in one but not the other type of nucleus. These genes are referred to as resistant genes. There were fewer genes resistant to reprogramming in transplanted ESCs compared with transplanted MEFs (538 versus 424, Figure 1B), presumably reflecting the more permissive chromatin organization of the pluripotent cell (Meshorer et al., 2006). A heatmap showing relative expression of resistant genes before and after nuclear transfer shows that resistance is associated with lower expression in the corresponding donor cells prior to transplantation (Figures 1C and 1D). This suggests that resistance originates from epigenetic mechanisms established in the somatic cell to ensure stable gene silencing, safeguarding cell identity. In agreement with this idea, gene ontology analysis of resistant genes indicates enrichment for functions related to transcription, development, and germ cell and reproduction (Figure 1E; Table S2). These functional categories may reflect the oocyte’s transcriptional activity toward an accumulation of factors necessary for the development of the oocyte and of the future embryo. Additionally, the Kyoto Encyclopedia of Genes and Genomes (KEGG) pathways enriched among resistant genes include cancer and tight junction in MEFs and cell signaling in ESCs (Figure 1F; Table S2). This suggests that the genes related to these functions are under a particularly strong repressive regime in the corresponding donor cell, leading to resistance to the reprogramming activity of the oocyte.

We conclude that, in mouse fibroblasts, a set of 538 genes, enriched for genes with early embryo/germ cell-related functions, is resistant to the reprogramming activity of the oocyte.

### Epigenetic Features of Resistant Genes

**Figure 2:**
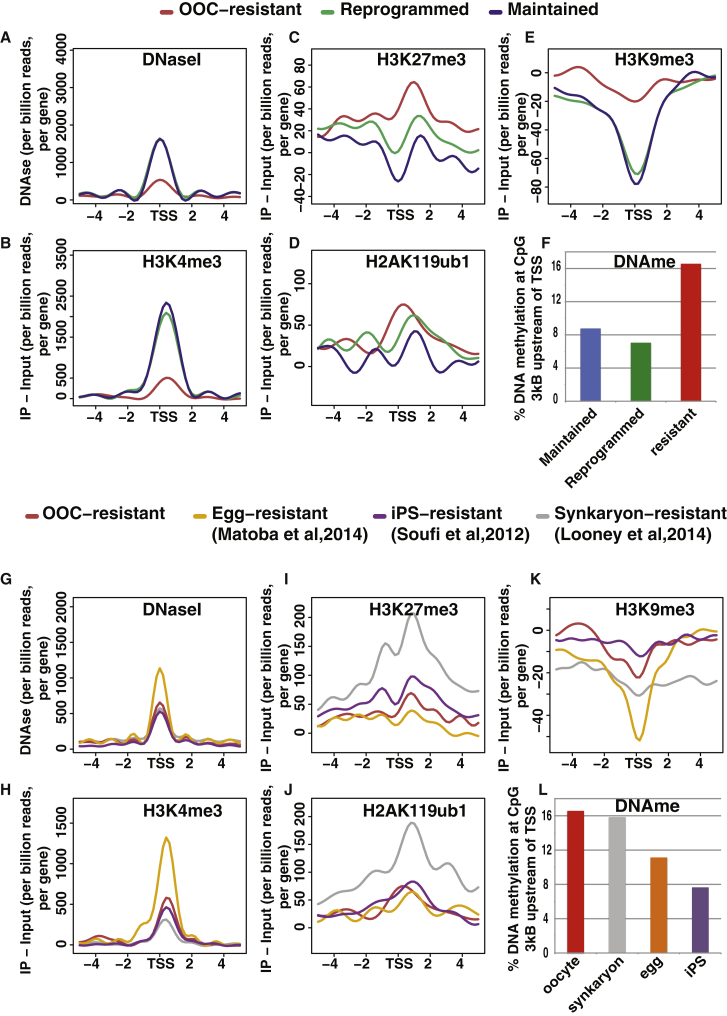
Epigenetic Configuration of MEF Genes Resistant to OOC-Mediated Transcriptional Reprogramming (A–E) Shown is the (A) DNase I sensitivity (Yue et al., 2014), (B) H3K4me3 (Kaneda et al., 2011), (C) H3K27me3 (Kaneda et al., 2011), (D) H2Aub (this study), and (E) H3K9me3 (Bulut-Karslioglu et al., 2014) metaplot analysis ±5 kB around the TSSs, in MEF, of genes with reprogrammed (green), maintained (blue), and resistant (red) expression following nuclear transfer. (F) DNA methylation in the promoter region (3 kb upstream TSS) of reprogrammed, maintained, or resistant genes is shown as the percentage of CpG showing >95% methylation (data from Reddington et al., 2013). (G–K) Shown is the (G) DNase I sensitivity (Yue et al., 2014), (H) H3K4me3 (Kaneda et al., 2011), (I) H3K27me3 (Kaneda et al., 2011), (J) H2Aub (this study), and (K) H3K9me3 (Bulut-Karslioglu et al., 2014) metaplot analysis ±5 kB around the TSSs of genes showing resistance in OOC-NT (red, from this study), mouse egg-NT (orange, from Matoba et al., 2014), transcription factor-induced reprogramming (purple, from Soufi et al., 2012), and cell fusion (gray, from Looney et al., 2014). (L) DNA methylation in the promoter region (3 kb upstream TSS) of genes resistant in OOC-NT, mouse egg-NT, transcription factor-induced reprogramming, and cell fusion is shown as the percentage of CpG showing >95% methylation (data from Reddington et al., 2013). See also Figure S2.

To understand how resistance is achieved, we first investigated what type of epigenetic features characterize the chromatin at the transcription start sites (TSSs) of resistant genes in the donor MEFs. We first compared resistant genes to genes whose expression we previously identified as maintained or successfully reprogrammed in MEF nuclei transplanted to oocytes (Jullien et al., 2014). As expected, on average, resistant genes exhibited a closed chromatin configuration when compared to maintained and reprogrammed genes, as shown by DNaseI sequencing (DNaseI-seq) (Yue et al., 2014) (Figure 2A). Resistant genes also were associated with low levels of H3K4me3, a histone modification associated with active transcription (Kaneda et al., 2011) (Figure 2B). By contrast, repressive marks H3K9me3 (Bulut-Karslioglu et al., 2014), H3K27me3 (Kaneda et al., 2011), and H2AK119ub1 all exhibited higher levels around the TSSs of resistant genes than around maintained or reprogrammed genes (Figures 2C–2E). Lastly, we observed that restricted genes exhibited a higher level of methylated CpG than maintained or reprogrammed genes (Figure 2F; Figure S2). These observations confirm that resistant genes have, as a whole, a different epigenetic configuration than genes that are readily reprogrammed or continuously expressed after nuclear transfer.

We further characterized the resistant gene state by asking whether resistance, as shown in the *Xenopus* oocyte nuclear transfer assay, bears any similarity, at the epigenetic level, with genes showing transcriptional resistance in other reprogramming assays. We compared the set of oocyte reprogramming-resistant genes identified here (Ooc resistant) to genes identified as resistant to reprogramming following transplantation to the mouse egg (egg resistant; Matoba et al., 2014), transcription factor-induced pluripotency (iPS resistant; Soufi et al., 2012), or cell fusion (Synkaryon resistant; Looney et al., 2014). Metaplot analysis indicates that, similar to Ooc-resistant genes, genes resistant to reprogramming in iPS, egg-NT, and cell fusion also showed reduced chromatin accessibility and enrichment for repressive histone marks when compared to successfully reprogrammed genes (Figures 2G–2L). This suggests that the epigenetic mechanisms conferring resistance to reprogramming are similar in these reprogramming systems.

In conclusion, we found that the Ooc-resistant genes have an epigenetic makeup that is similar to that of genes resistant to reprogramming in other systems and that distinguishes them from successfully reprogrammed genes. We next sought to investigate whether these epigenetic marks mediate resistance to transcriptional reprogramming.

### Overexpression of Chromatin Modifiers Alleviates Resistance

**Figure 3:**
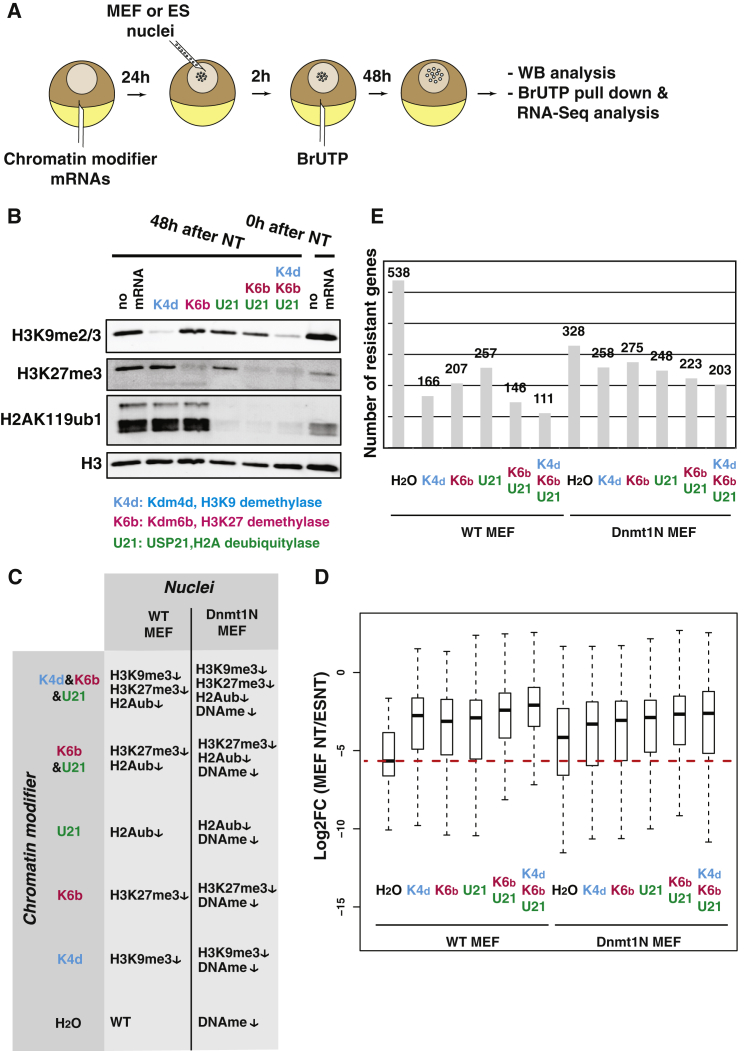
Chromatin Modifier Overexpression Alleviates Resistance to Transcriptional Reprogramming (A) Schematic representation shows the nuclear transplantation strategy used to test the sensitivity of resistant genes to chromatin modifiers. (B) Western blot analysis of modified histones on chromatin recovered 48 hr after nuclear transplantation to oocyte expressing single or multiple chromatin modifiers is shown. (C) Summary shows the 12 chromatin configurations tested in the OOC transcriptional reprogramming assay. (D) Boxplot shows the log fold change between resistant gene expression in MEF-NT in various chromatin configurations to that of ES-NT. (E) Number of resistant genes as judged by differential gene expression analysis between RNA-seq data from ES-NT and MEF-NT in various chromatin configurations (FDR < 0.05, two experiments, n = 32 NT samples per conditions). See also Figure S3 and Table S3.

To alter the chromatin of transplanted nuclei, we pre-injected oocytes with mRNAs encoding chromatin modifiers either alone or in combination (Figure 3A). Based on the chromatin features of resistant genes described above, we used chromatin modifiers targeting histone modifications involved in transcriptional repression: Kdm4d (H3K9 demethylase; Shin and Janknecht, 2007), Kdm6b (H3K27 demethylase; Agger et al., 2007), and USP21 (H2AK119 deubiquitylase; Nakagawa et al., 2008). We also used a combination of USP21 and Kdm6b in order to erase repressive histone marks associated with polycomb repressive complexes 1 and 2, respectively. Lastly, we combined the removal of polycomb complex repressive marks with that of H3K9 methylation (co-expression of USP21, Kdm6b, and Kdm4d). Then 24 hr after mRNA injection, nuclei were transplanted to the oocytes, and, another 48 hr after nuclear transfer, the transplanted nuclei were recovered and analyzed by western blot analysis. The overexpression of chromatin modifiers led to an efficient removal of histone modifications from the transplanted nuclei (Figure 3B; Figure S3).

Using this chromatin modification strategy, we transplanted nuclei from wild-type (WT) or DNA methylation-deficient fibroblasts (Dnmt1N MEF; Jackson-Grusby et al., 2001, Lande-Diner et al., 2007) to oocytes expressing chromatin modifiers, and we analyzed transcriptional reprogramming by RNA-seq. This allowed us to determine the expression of resistant genes from two types of transplanted chromatin (with or without DNA methylation) in six different histone modification configurations (Figure 3C). We observed that chromatin modifier expression increased the average expression level of resistant genes, in agreement with the role of the targeted histone modifications in gene repression (Figure 3D). The observed increase in expression was associated with a loss of resistance in a large proportion of resistant genes for any given treatment, as judged by the loss of differential expression between ESCs and treated MEFs 48 hr after nuclear transfer (Figure 3E; Table S3). Of all single treatments, Kdm4d was the most effective and the loss of DNA methylation was the least effective (Figure 3E, 166 and 328 resistant genes left, respectively). The greatest effect was found with the combination of Kdm4d, Kdm6b, and USP21 on chromatin with normal DNA methylation (Figure 3E). Interestingly, in the context of DNA hypomethylation, the expression of chromatin modifiers had a less pronounced effect.

We conclude that overexpression of chromatin modifiers generally decreases the resistance to transcriptional reprogramming both in the presence and absence of DNA methylation.

### A Unique Pattern of Resistant Gene Sensitivity to Chromatin Modifier Treatment

**Figure 4:**
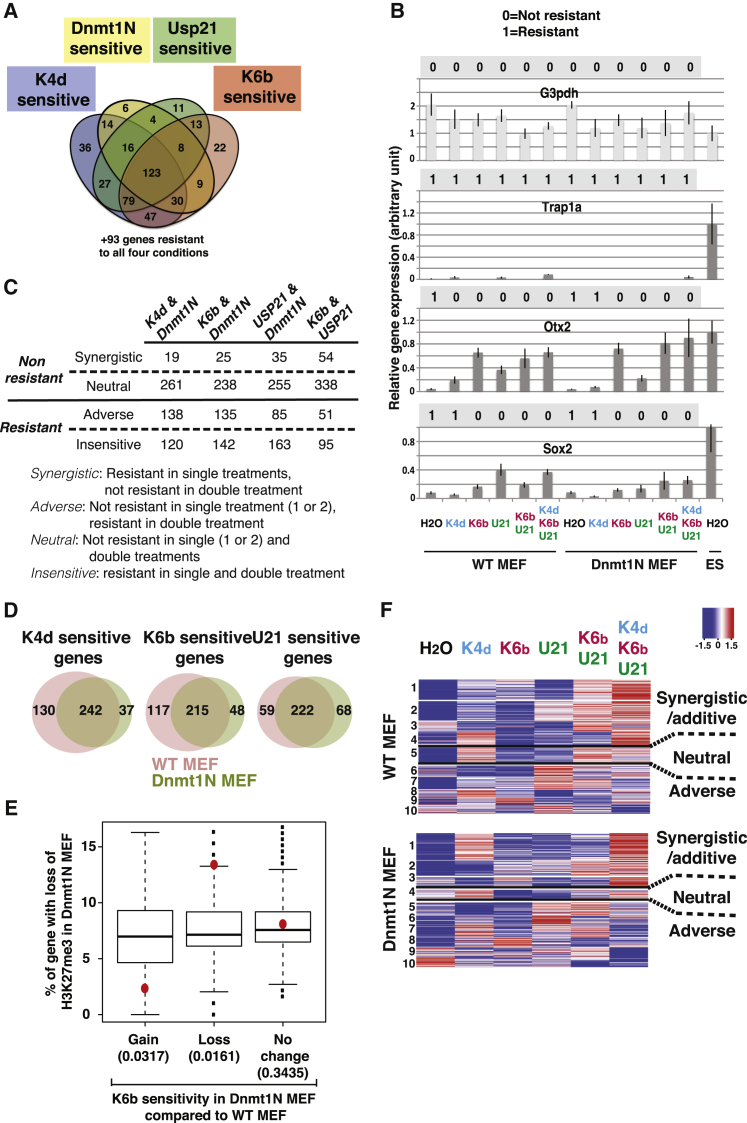
Multiple Chromatin Modifier Combinations Affect Resistance at a Single-Gene Level (A) Venn diagram shows the number of genes losing resistance upon four individual chromatin modifier treatments. (B) Example of digital signature of resistance obtained by differential gene expression analysis for a non-resistant (*G3pdh*) and three resistant (*Trap1A*, *Otx2*, and *Sox2*) genes. The digital signature indicates whether a gene is resistant (shown as 1 when the gene is differentially expressed in MEF-NT compared to ES-NT) or loses resistance (shown as 0, when the gene is not differentially expressed in MEF-NT compared to ES-NT) in each of the 12 conditions tested. The bar graphs show qRT-PCR analysis of gene expression (mean ± SEM of four samples of eight oocytes NT). (C) The numbers of genes for which a synergistic, neutral, adverse, or insensitive effect is observed when combining two chromatin modifier treatments are shown. (D) Venn diagram shows the number of genes sensitive to a histone modifier (Kdm4d, Kdm6b, or USP21) in nuclei with normal (WT) or hypomethylated DNA (Dnmt1N). (E) Loss of resistant gene sensitivity to Kdm6b in DNA-hypomethylated MEF correlates with loss of H3K27me3. The y axis indicates the number of genes that lose H3K27me3 in MEF with hypomethylated DNA compared to WT MEF. Resistant genes are split according to change in sensitivity to Kdm6b after NT when comparing WT MEF to MEF with hypomethylated DNA (red dot). The boxplot shows the background distribution of H3K27me3 change when sampling 10,000 times for a random set of genes of the same size as the resistant gene subset tested. The number in parentheses indicates a p value generated by calculating the proportion of the random background that has a more extreme value than the observed percentages from each of the three groups. (F) Heatmap illustrating the change in gene expression in normal (WT MEF nuclei) or hypomethylated DNA (Dnmt1N MEF nuclei) upon expression of histone modifiers. Ten clusters representing the main trend of gene expression change were selected based on k-means clustering of expression data from RNA-seq analysis (RPKM). The trend of change in gene expression in these ten clusters is shown in the heatmap as a *Z* score. See also Table S4.

Each chromatin modifier treatment was affecting the expression of a large fraction of resistant genes (Figure 3E). This suggests that, at a single-gene level, resistance can be alleviated by more than one type of treatment. To investigate the sensitivity of resistance to multiple treatments, we focused first on the effect of single chromatin modifier treatment, and we evaluated how often overexpression of Kdm4d, Kdm6b, and USP21 as well as inactivation of Dnmt1 could affect the expression of the same resistant genes (Figure 4A). First, we observed that only 93 resistant genes were not affected by any of these four treatments. The remaining genes (445/538) lost resistance in at least one of the treatments, and the majority was affected by more than one treatment. Interestingly, a very large number of resistant genes (123) were upregulated in all four individual treatments. Altogether, these observations suggest that, at a single-gene level, multiple silencing mechanisms contribute resistance to transcriptional reprogramming by the oocyte factors.

To investigate further the relationship between resistance and chromatin modifiers at a single-gene level, we classified resistant genes according to their resistance to transcriptional reprogramming in all 12 conditions tested (listed in Figure 3C). For each resistant gene, differential expression analysis at 48 hr after nuclear transfer between ESC nuclei and MEF nuclei was used to determine whether this gene maintained or lost resistance in the various treatment used. This can be represented as a digital signature of resistance. When a gene was still differentially expressed upon a given treatment in MEF-NT compared to ES-NT (LogFC [MEF/ES] < 0 and FDR < 0.05), it was assigned a value of 1, indicating that resistance was maintained in that condition. If by contrast there was no differential expression of the gene between treated MEF-NT and ES-NT, it was assigned a value of 0, indicating that resistance was lost in that condition. An example of this signature is shown for three resistant genes, *Trap1a*, *Sox2*, and *Otx2*, and for one non-resistant gene, *G3pdh* (Figure 4B). When analyzed by qRT-PCR, an increase in gene expression was detected for all treatments where differential gene expression analysis also identified a loss of resistance. For all 538 resistant genes, such a digital signature was generated (Table S4). This analysis showed that the epigenetic features tested here account for a significant part of the resistance to transcriptional reprogramming by the oocytes, as only 47 genes (8.7% of all resistant genes) remained resistant when considering all conditions tested. We identified a very large number of digital signature types (232 for 538 genes), indicating that the pattern of sensitivity to chromatin modifier treatment is almost unique to each resistant gene.

### Synergistic, Neutral, and Adverse Effects of a Combination of Chromatin Modifiers

We next sought to investigate the effect of combining chromatin modifier treatments on loss of resistance. We compared the use of two simultaneous treatments versus single treatments. The effects seen fell into one of three classes.

First, we observed a neutral effect, when the combined treatments led to the same effect on resistance as the single treatments. This took place when loss of resistance was seen at least in one of the single treatments, and this loss was still seen when combining the treatments (neutral, Figure 4C). We also found instances where the gene remained resistant in both the single and combined treatments (insensitive, Figure 4C). This lack of additional effect of combining chromatin modifiers was by far the most common (neutral and insensitive accounted for >70% of resistant genes in all combinations tested).

Additionally, we observed cases of synergistic effects when loss of resistance was only seen in the double treatments. This effect was not so common when combining loss of DNA methylation to histone modifier. However, USP21 and Kdm6b synergistically removed resistance from about 10% of resistant genes (54/538). This suggests that the repressive histone marks targeted by these enzymes (H2AK119ub1 and H3K27me3, associated with polycomb repressive complexes 1 and 2 [PRC1 and PRC2], respectively) independently ensured repression of these genes.

Surprisingly, we observed numerous instances of adverse effect, when a gene lost resistance in at least one individual treatment but remained resistant when the treatments were combined (Figure 4C). This happened particularly when a gene lost resistance upon histone modifier treatment only in the context of normal DNA methylation, but not in the context of chromatin with reduced DNA methylation (Figure 4D). This adverse effect of DNA methylation loss on resistance sensitivity to chromatin modifier suggests that inactivation of Dnmt1 in the cultured cell induces changes in the distribution of repressive histone modification around resistant genes in the donor nuclei.

To investigate this possibility, we analyzed the relationship between the distribution of H3K27me3 in WT and DNA hypomethylated cells and the sensitivity of resistant genes to the H3K27 demethylase Kdm6b following nuclear transfer. Previous studies have shown significant changes in the distribution of H3K27me3 upon loss of DNA methylation (Reddington et al., 2013). Decrease in DNA methylation level leads mostly to a spreading of H3K27me3 to a larger part of the genome without an overall increase in H3K27me3 in the cell, resulting in H3K27me3 loss from numerous genomic locations. We observed that loss of H3K27me3 upon reduction of DNA methylation was more pronounced on resistant genes that lost sensitivity rather than maintained sensitivity to Kdm6b, when comparing the reprogramming of DNA hypomethylated nuclei to that of WT nuclei (Figure 4E). This suggests that loss of Kdm6b sensitivity in hypomethylated DNA nuclei is indeed mediated by a redistribution of H3K27me3 in the donor nuclei.

Digital signature analysis identified genes that show the same pattern of loss of resistance across multiple treatments (Table S4). However, the change in gene expression that underlies this loss of resistance could vary greatly between treatments and is not reflected in this categorization. For example, the loss of resistance for *Otx2* in the presence of Kdm4d and Kdm6b corresponded to an increase in gene expression over control conditions of 4- and 15-fold, respectively, as judged by qRT-PCR analysis (Figure 4B). To take into account more subtle changes in gene expression underlying loss of resistance, we clustered resistant gene expression levels across all treatments (Figure 4F). We reduced the number of patterns of resistance sensitivity to treatments from the initial 232 types identified by digital signature analysis (Table S4) to ten main groups (Figure 4F). When taking into account the scale of the change in gene expression, we could further reduce the complexity of pattern observed by grouping resistant genes according to their trend of expression in response to chromatin modifier treatments. This analysis indicated that the effect of a combination of chromatin modifiers on resistance is rarely neutral and that synergistic/additive and adverse effects are much more common.

### Loss of Resistance upon Usp21 Expression Correlates with Enrichment for H2Aub on the Affected Genes

Given that, at a single-gene level, resistance often can be removed by the expression of more than one type of chromatin modifier, this raises the question of the mechanism of action of these factors (Figure 4A). Indeed, although the reprogramming system described here is independent of replication and is directly mediated by oocyte factors, it does not exclude the possibility that the chromatin modifiers act on transcription independently of their activity against their cognate chromatin targets. In fact, targets besides the classical histones, such as transcription factors, have been found to be affected by chromatin modifiers (Carr et al., 2015).

**Figure 5:**
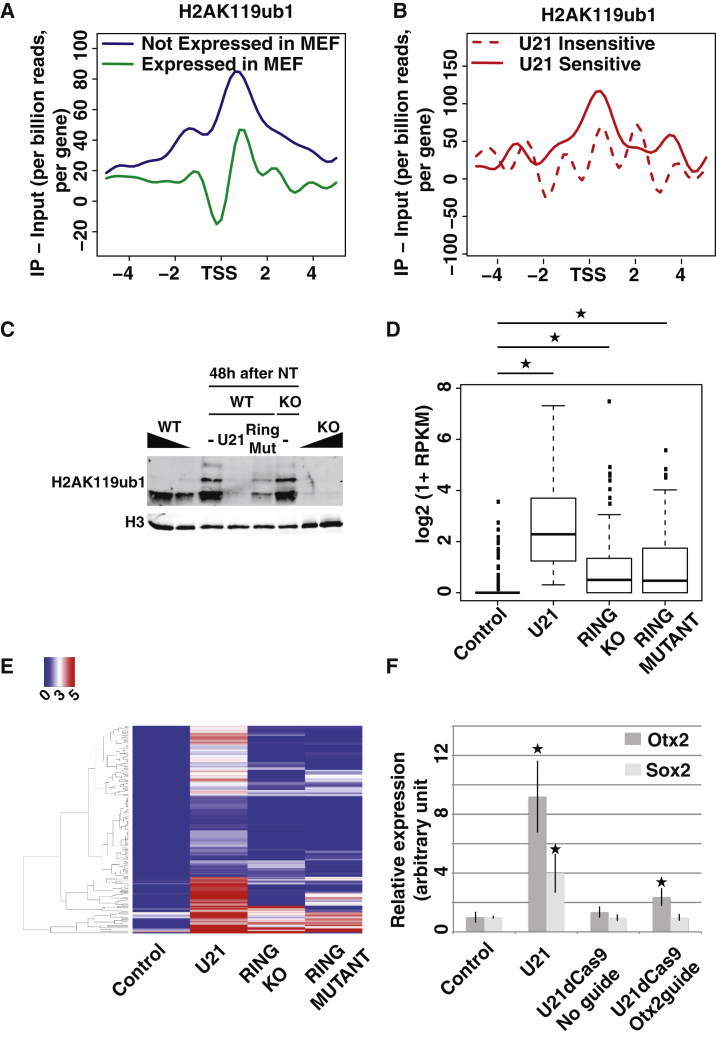
USP21 Removes Resistance through Deubiquitylation of H2A (A) Metaplot analysis in MEF of H2AUb ChIP-seq ±5 kb around the TSSs of genes expressed (green) or not expressed (blue) in MEF is shown. (B) Same as (A) but resistant genes are split according to sensitivity (red line) or insensitivity (red dashed line) to USP21. (C) WB analysis of H2AK119ub1 level in WT or *Ring1a&b* KO-transplanted nuclei 0 and 48 hr after nuclear transfer. Nuclei were transplanted in control oocyte or in oocyte overexpressing USP21 or catalytically inactive *Xl*Ring1b (Ring Mut). (D) Boxplot shows expression of the set of genes upregulated in WT MEF transplanted to USP21 compared to WT MEF transplanted to control oocytes (★p value < 10^−11^, Welch two-sample t test). (E) Heatmap showing unsupervised hierarchical clustering of genes upregulated in WT MEF transplanted to USP21-expressing oocyte compared to control oocytes in WT MEF transplanted to control oocytes, to USP21-expressing oocytes, to *XlRing1b* mutant-expressing oocytes, and in *Ring1a&Ring1b* KO MEF transplanted to control oocytes. Expression value is shown as log2(1 + RPKM). (F) dCas9-USP21 targeted to the *Otx2* promoter decreases *Otx2* resistance to transcriptional reprogramming. The qRT-PCR analysis of *Otx2* and *Sox2* gene expression 48 hr after MEF transplantation to oocytes injected with water (control), USP21 RNA, or USP21-dCas9 RNA, with or without *Otx2* gRNAs mix, is shown (★p value < 0.05, Student’s t test; mean ± SEM of four experiments). See also Figure S4 and Table S5.

To investigate if chromatin modifiers affect resistance through the removal of repressive marks from chromatin, we first analyzed whether loss of resistance after expression of a given chromatin modifier correlates with the presence of its target epigenetic mark on the resistant genes. Because its chromatin modifier activity is less well characterized than that of Kdm4d and Kdm6b, we focused on the characterization of USP21 effect on resistance. We first investigated the distribution of H2AK119ub1 by chromatin immunoprecipitation sequencing (ChIP-seq). H2AK119ub1 was more abundant around the TSSs of genes that were not expressed rather than expressed in cultured MEFs, in accordance with the repressive function of this histone modification (Zhou et al., 2008) (Figure 5A). When focusing on resistant genes, we observed a relative H2AK119ub1 enrichment on USP21-sensitive genes compared to USP21-insensitive genes in donor nuclei (Figure 5B). The fact that high levels of H2Aub were present on USP21-sensitive genes was consistent with the idea that USP21 suppresses resistance through deubiquitylation of H2AK119 on this set of gene. We next sought to test this mechanism by complementary approaches.

### USP21 Removes Resistance through the Removal of H2A Ubiquitylation

We first reduced the level of H2AK119ub1 on transplanted chromatin *after* nuclear transfer. This was achieved either by overexpressing the deubiquitylase USP21, as described above, or by overexpressing a catalytically inactive form of XlRing1b, the E3 ligase responsible for H2A ubiquitylation by the PRC1 complex (de Napoles et al., 2004). Similar to USP21 overexpression, Ring1B mutant overexpression reduced the level of H2AK119ub1 on transplanted chromatin 48 hr after transplantation (Figure 5C). Second, we reduced H2AK119ub1 level in the donor nuclei *prior* to nuclear transfer using Ring1a&Ring1b knockout (KO) MEF (Endoh et al., 2012). In that case, the transplanted nuclei were initially devoid of H2AK119ub1. However, 48 hr after nuclear transfer, they regained levels of H2AK119ub1 similar to those of WT nuclei (Figure 5C).

We then used RNA-seq analysis to investigate the effect of these three treatments on gene expression following nuclear transfer. USP21 overexpression, XlRing1B mutant overexpression, and transplantation of Ring1a/b KO nuclei resulted in an increased expression over control nuclear transfer of 165, 73, and 79 genes, respectively (Table S5). We first noted a correlation between the extent of H2AK119ub1 level decrease and the number of upregulated genes, suggesting that the effect observed on gene expression was indeed dictated by the effect of the treatments on H2AK119ub1 level. Importantly, the genes upregulated in conditions where Ring1a/b KO nuclei were transplanted or XlRing1b mutant was overexpressed were significantly enriched for the genes that also were upregulated by USP21 overexpression (22/79 and 13/73, respectively; p value < 10^−10^, hypergeometric test). Although statistically significant, the overlap between these gene sets is relatively small, presumably because of the differential effect of the corresponding treatment on H2AK119ub1 level during the course of the experiment (see above). Additionally, the genes that were upregulated in MEF nuclei transplanted to USP21-expressing oocytes compared to control oocytes also showed significantly higher expression in transplanted Ring1a&b KO nuclei or in WT nuclei transplanted to oocytes overexpressing XlRing1b mutant (Figure 5D). Lastly, the genes that were upregulated upon USP21 overexpression showed a similar pattern of upregulation in the two other treatments reducing H2AK119ub1 level (Figure 5E).

We observed that three ways of interfering with H2AK119ub1 led to the upregulation of a similar set of genes in transplanted nuclei. We conclude that USP21 overexpression removes resistance mostly through its H2A deubiquitylation activity.

To further test whether USP21 acts through chromatin deubiquitylation of H2A, we targeted USP21 to one resistant gene rather than the whole nuclear chromatin. We fused USP21 to dCas9 in order to direct USP21 to the promoter region of the resistant gene *Otx2* using small guide RNAs (sgRNAs) (Figure S4). We found a significant loss of *Otx2* resistance to transcriptional reprogramming following nuclear transfer to oocytes expressing USP21dCas9 and guide RNAs targeting the *Otx2* promoter, but not in the absence of guide RNAs (Figure 5F). This correlates with a guide RNA-dependent deubiquitylation of H2A at the *Otx2* promoter (Figure S4C). Importantly, in the conditions used, global levels of H2Aub were not decreased, as shown by western blot (WB) (Figure S4A) and ChIP-qPCR on repeat elements of the genome (major satellites and intracisternal A particles [IAPs], Figure S4C). We conclude that a localized decrease in H2Aub at the promoter is sufficient to reduce resistance following nuclear transfer.

### USP21 Improves Transcriptional Reprogramming of Cloned Mouse Two-Cell Embryos

**Figure 6:**
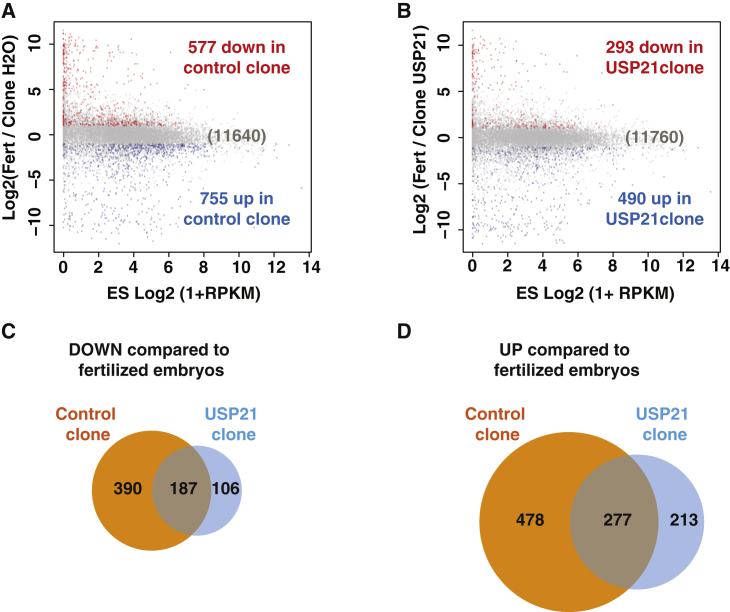
USP21 Improves Transcriptional Reprogramming of Two-Cell-Stage Cloned Mouse Embryos (A) MA plot showing log fold change in gene expression between fertilized embryos and ES clone embryos (logFC, y axis) against the log of gene expression in cultured ES (logCPM, x axis). Differentially expressed genes are shown in red and blue for genes downregulated and upregulated in clones compared to fertilized embryos, respectively (FDR < 0.05, three experiments). (B) Same as (A) except that USP21 was overexpressed in the cloned embryos. (C and D) Venn diagram indicating the overlap between the downregulated genes (C) and upregulated genes (D) in control and USP21 clones. See also Figure S5 and Tables S6 and S7.

We next asked whether USP21 also could alleviate resistance to transcriptional reprogramming after mouse nuclear transfer to enucleated MII oocytes. We performed cloning experiments in the mouse, in which mouse ESC (mESC) nuclei were transplanted to enucleated eggs. We let such reconstituted embryos develop to the two-cell stage, when zygotic gene activation occurs, and we analyzed gene expression by RNA-seq (Figure 6A). As a control, we used two-cell-stage embryos obtained by in vitro fertilization. We identified 1,332 genes misregulated in cloned embryos compared to fertilized embryos (577 downregulated genes and 755 upregulated genes, FDR < 0.05 and |logFC| > 1; Table S6), a number similar to what has been described in two-cell-stage embryos cloned from mouse fibroblast nuclei (Matoba et al., 2014). We then asked whether the gene misregulation observed in the cloned embryos could be reduced by USP21 treatment. To that end, cloned embryos were reconstructed by transplanting ESC nuclei to enucleated egg pre-injected with USP21 mRNA injection. At the two-cell stage, the chromatin of these embryos exhibited a reproducible reduction of H2AK119ub1 level (Figure S5). RNA-seq analysis indicated 293 downregulated genes and 490 upregulated genes in USP21 clones compared to in vitro fertilization (IVF) embryos (Figure 6B). This corresponds to a reduction in the number of downregulated and upregulated genes between USP21 clones and control clones of ∼50% and ∼35%, respectively (Figures 6C and 6D).

We conclude that resistance to transcriptional reprogramming at zygotic gene activation in cloned mouse embryos also can be reduced by the expression of USP21.

## Discussion

Interference with several epigenetic pathways, such as those involving repressive methylation of histones (Soufi et al., 2012, Onder et al., 2012), DNA methylation (Mikkelsen et al., 2008), or histone deposition (Cheloufi et al., 2015, Gaspar-Maia et al., 2013, Pasque et al., 2012), all can lead to an improvement in iPS reprogramming efficiency. In these experiments, however, it is not clear whether the increase in efficiency is mechanistically linked to a direct effect of chromatin modifiers on the transcription of genes resistant to reprogramming. Additionally, it is not known whether different ways of improving reprogramming through chromatin-based interference act on similar or independent pathways, as they have not been systematically combined, presumably because of the detrimental effect of doing so on cell viability. Indeed, interference with chromatin chaperones together with histone methylase or components of PRC1 and PRC2 have been reported to have adverse effects in iPS experiments, precluding the analysis of treatment combinations (Cheloufi et al., 2015, Onder et al., 2012).

Our oocyte-reprogramming assay allowed us to circumvent this problem and measure the direct effect of removing chromatin repressive marks, alone or in combination, on gene transcription. Strikingly, challenging the stability of the repressed state through nuclear transfer reveals that many of the resistant genes are directly sensitive to interference with more than one epigenetic repressive pathway. This observation suggests that the stabilization of gene expression pattern involves the establishment of multiple layers of epigenetic repression on a subset of genes critical for safeguarding the identity of a cell. This is in agreement with mechanisms described for gene silencing during cell differentiation. For example, silencing of the pluripotency gene Oct4 during stem cell differentiation entails the deacetylation of histone followed by the methylation of H3K9 and, eventually, DNA methylation (Epsztejn-Litman et al., 2008, Feldman et al., 2006). Similarly, the inactivation of X chromosome in female cells during early embryogenesis in the mouse involves the concerted action of multiple repressive pathways, including long noncoding RNAs, H3K27 methylation, histone variant deposition, and DNA methylation (Heard and Disteche, 2006). Therefore, loss of resistance may require the reversal of multiple layers of repressive features added to genes undergoing silencing as cell differentiate.

Whether histone modifications are themselves functionally implicated in the regulation of transcription is not well defined (Henikoff and Shilatifard, 2011). Most functional tests are based on the inactivation of histone-modifying complex. As a consequence, it is difficult to disentangle the observed effect resulting from loss of the histone mark to that resulting from loss of the histone-modifying complex. An exception to this is the use of mutant histone that cannot be methylated on a given lysine residue (Herz et al., 2014). *Drosophila* expressing such histone mutant, H3K27M, exhibits a phenotype that mimics the loss of PRC2, directly implicating methylation of H3K27 in the observed effect. A more conflicting picture emerges from the use of cells engineered to have a PRC1 devoid of H2A ubiquitylation activity. Some reports suggest that H2Aub is not required for the repression of transcription, whereas others found that it is (Endoh et al., 2012, Illingworth et al., 2015, Pengelly et al., 2015). We observe that three independent ways of globally interfering with H2AK119ub1 as well as targeted removal of this histone modification are able to reduce resistance to direct transcriptional reprogramming. This indicates that H2AK119ub1 itself contributes to the observed resistance. In mouse ESCs, H2A deubiquitylases USP16 and USP21 are required for proper differentiation, suggesting that modulation of H2AK119ub1 genomic distribution is necessary for efficient change in cell fate (Jin et al., 2016, Yang et al., 2014). It is, therefore, possible that overexpression of a H2A deubiquitinase in cultured cells could also improve the efficiency of cell fate conversion in induced pluripotency.

To improve gene reactivation for efficient reprogramming, interference with multiple repressive pathways might be required. However, we also observe that a combination of chromatin modifiers quite often has an adverse effect on resistance removal. Indeed, it appears that some of the marks targeted can have repressive functions on some resistant genes while being required for proper reactivation of others. In that regard, refined strategies involving the targeting of epigenetic modifiers to resistant genes according to their sensitivity should provide a better transcriptional reprogramming outcome (Zalatan et al., 2015). To achieve this, further investigations are needed to delineate precisely which part of the genome requires modification in order to relieve resistance. In particular it would be important to more generally test whether histone modifications impose resistance through large domains or through localized deposition, as previous studies have correlated the resistant state to the presence of modified histones on large stretches of the genome (Matoba et al., 2014, Wen et al., 2009). Lastly it would be important to compare the resistance to transcriptional reprogramming in donor cells from different degrees of differentiation in order to establish whether the mechanisms stabilizing cell identity change as cells differentiate.

## STAR★Methods

### Key Resources Table

**Table:** 

REAGENT or RESOURCE	SOURCE	IDENTIFIER
**Antibodies**		

Rabbit anti H2AK119ub1	Cell Signaling	8240; RRID: AB_10891618
Mouse anti H3K9me3/2	Cell Signaling	5327
Rabbit anti H3K27me3	Cell Signaling	9733; RRID: AB_2616029
Rabbit anti H3	Abcam	ab18521; RRID: AB_732917
Mouse anti-BrdU Agarose beads	Santa-Cruz	sc-32323 AC; RRID: AB_626766
Mouse anti H4	Abcam	ab31830; RRID: AB_1209246
Mouse anti-HA	SIGMA	H3663; RRID: AB_262051
Rabbit anti-HA	Abcam	ab9110; RRID: AB_307019
Goat anti mouse alexa 680	Thermo Fisher	A21058; RRID: AB_2535724
Goat anti rabbit alexa 800	Thermo Fisher	A32735; RRID: AB_2633284
Donkey IgG anti-Rabbit IgG (H+L)-Alexa Fluor 488	Jackson ImmunoResearch	711-545-152; RRID: AB_2313584

**Chemicals, Peptides, and Recombinant Proteins**		

LIF	chemicon	ESG 1107
LIF	Cell guidance	GFM200
GMEM BH-21	GIBCO	21710-025
DMEM	GIBCO	31966-021
Fetal Calf serum	GIBCO	10439-024
Fetal Calf serum	FISCHER	10117272
PD0325901 (MEK inhibitor)	TOCRIS	4192
CH99021 (GSK3 inhibitor)	Medchemexpress Europe	CHIR-99021
Non-essential amino acid	GIBCO	11140-035
Sodium Pyruvate	GIBCO	11360-039
Betamercaptoethanol	GIBCO	31350-010
Ethyl 3-aminobenzoate methanesulfonate (MS222)	SIGMA	E10521
Liberase	ROCHE	05401127001
BrUTP	SIGMA	B7166
Superase (RNase inhibitor)	AMBION	AM2696
Protease inhibitor cocktail (aebsf; aprotinin; bestatin; E-64; Leupeptin; Pepstatin)	SIGMA	SRE0055
pENTRY/D-TOPO Cloning Kit	Invitrogen	K2400-20
Gateway LR Clonase II Enzyme Mix	Thermo Fisher	11791-20
Phusion High Fidelity Polymerase	New England Biolabs	M0530
hyaluronidase	SIGMA	H4272
M2 medium	SIGMA	M7167
Cytochalasin B	SIGMA	C6762
Demelcocine	SIGMA	D7395
M16 medium	SIGMA	M7292

**Critical Commercial Assays**		

Ovation Single Cell RNA-Seq	Nugen	0342-32
TruSeq DNA Sample Prep Kit	Illumina	FC-121-2001
RNAeasy mini Kit	QIAGEN	74106
QIAquick PCR purification Kit	QIAGEN	28104
ARCTURUS PicoPure RNA isolation kit	Thermo Fisher	KIT0204
MEGAscript SP6 Kit	AMBION	AM1330
MEGAscript T7 Kit	AMBION	AM1334

**Deposited Data**		

RNA-seq&ChIP-seq	This paper	GEO: GSE87872
*Xenopus laevis* genome (JGI version 6.1)	*Xenopus* genome project	ftp://ftp.xenbase.org/pub/Genomics/JGI/Xenla6.1/
Images	This paper	http://dx.doi.org/10.17632/jzjspk58nv.1

**Experimental Models: Cell Lines**		

P53−/− MEF	Cedar Lab	Jackson-Grusby et al., 2001
P53−/− dnmt1N MEF	Cedar Lab	Jackson-Grusby et al., 2001
*Ring1A*^−*/*−^*;Ring1B*^*fl/fl*^*;Rosa26::CreERT2* MEF	Koseki Lab	Endoh et al., 2012
ES cells, derived from 129Sv/Pas blastocysts	This paper	N/A
C57BL/6 ES cells	EUCOMM repository	EPD0157_4_A11

**Experimental Models: Organisms/Strains**		

eight-week old C57/CBAF1 mice	Janvier Labs	B6CBAF1/JRj
Mature (3-6 year old) *Xenopus laevis Laevis* female	Nasco	LM00535MX

**Recombinant DNA**		

pCS2+ USP21 NLS-HA	This paper	N/A
pCS2+ KDM4D NLS-HA	This paper	N/A
pCS2+ Kdm6b (aa1025-1642) NLS-HA	This paper	N/A
pCS2+ xlRing1b I49A NLS-HA	This paper	N/A
pCS2+ USP21-dCas9- NLS-HA	This paper	N/A

**Sequence-Based Reagents**		

Guide otx2 promoter (Table S8)	This paper	N/A
qPCR primers (Table S8)	This paper	N/A
Superscript III reverse transcriptase	Invitrogen	18080093
Sybergreen	SIGMA	S9194

**Software and Algorithms**		

BWA 0.6.2-r126-TPX	Li and Durbin, 2009	https://sourceforge.net/projects/bio-bwa/files/
SICKLE 1.200	Joshi and Fass, 2011	https://github.com/najoshi/sickle
FASTX-TOOLKIT 0.0.13	Hannon lab	http://hannonlab.cshl.edu/fastx_toolkit/
samtools 0.1.8	Li et al., 2009	http://samtools.sourceforge.net.
BAMTOOLS 2.1.1	Barnett et al., 2011	https://github.com/pezmaster31/bamtools
cutadapt 1.0	Martin, 2011	https://pypi.python.org/pypi/cutadapt/1.0
TopHat version 2.0.6	Trapnell et al., 2009	https://ccb.jhu.edu/software/tophat/index.shtml
R version 3.2.4	R-Core-Team, 2016	https://www.R-project.org/
edgeR	Robinson et al., 2010	https://bioconductor.org/packages/release/bioc/html/edgeR.html
gplots package version 3.0.1	Warnes et al., 2016	https://cran.r-project.org/web/packages/gplots/index.html
ImageJ		https://imagej.nih.gov/ij/

### Contact for Reagent and Resource Sharing

Further information and requests for resources and reagents should be directed to and will be fulfilled by the Lead Contact, Dr J. Jullien (j.jullien@cam.ac.uk).

### Experimental Model and Subject Details

#### Cell lines

Mouse fibroblasts (p53−/−; p53−/− Dnmt1N; and *Ring1A*^−*/*−^*;Ring1B*^*fl/fl*^*;Rosa26::CreERT2;SV40) were cultivated in* Dulbecco’s Modified Eagle Medium *(DMEM), 5% Fetal Calf serum on gelatin coated dish.* C57BL/6 ES cells were cultured without feeders on gelatin coated dish in the presence of 1000U/ml leukemia inhibitory factor (LIF, CHEMICON ESG 1107) in Glasgow modification of Eagles medium (GMEM BH-21, GIBCO 21710-025) containing 15% fetal calf serum (GIBCO 10439-024) supplemented with 1 μM MEK inhibitor PD0325901 (TOCRIS 4192), 3 μM GSK3 inhibitor CH99021 (Medchemexpress Europe, CHIR-99021), 0.1mM non-essential amino acid (GIBCO 11140-035), 10mM sodium pyruvate (GIBCO 11360-039), and 0.1mM betamercaptoethanol (GIBCO 31350-010). For nuclear transfer to mouse egg, ES cell CK35, were derived from 129Sv/Pas blastocysts (generous gift from Pasteur Institute Mouse Functional Genetics Unit). Cells were cultured according to standard conditions in flasks, on gelatin, in DMEM (GIBCO 31966-021) supplemented with 15% fetal calf serum (FISHER 10117272) and LIF (1000 U/ml, Cell Guidance: GFM200).

#### *Xenopus laevis* laevis husbandry

Mature *Xenopus laevis* males and females were obtained from Nasco (901 Janesville Avenue, PO Box 901, Fort Atkinson, WI 53538-0901; https://www.enasco.com/xenopus). Our work with *Xenopus laevis* is covered under the Home Office Project License PPL 70/8591 and frog husbandry and all experiments were performed according to the relevant regulatory standards. Animals were kept in a Marine Biotech recirculating system at a density of one adult/3l, with 10% water change per day. Water was sequentially filtered with mechanical pad sump filter, nitrifying bacteria filter, mechanical canister filter, carbon filter, and UV sterilized. Water quality parameters were as follow: conductivity 1500us; temperature 17-22°C; PH 6-8. Photoperiod was set to 12h ON/12h OFF. Frogs are fed twice per week with Royal Horizon 4.5mm pellets (skretting, https://www.skrettingfishfeeds.co.uk/). Unconsumed food was removed 10 min after the start of feeding.

#### *Mus musculus* husbandry

*Mus musculus* (strains C57CBA; males and females) were obtained from Janvier Labs (Route des Chênes Secs 53940 Le Genest-Saint-Isle France; https://www.janvier-labs.com) and housed at the UE IERP rodent resource center from INRA in Jouy en Josas (Unite Experimentale d’Infectiologie Experimentale des Rongeurs et Poissons). Animal care and handling at INRA is carried out according to European regulations on animal welfare. N. Beaujean has the authorization to work with laboratory animals from the French veterinary regulatory services (N°78–95) and the present work on mouse has been approved by the ethics committee (no. 12/123; Comethea Jouy-en-Josas/AgroParisTech). Animals are housed in groups of maximum 2-5 mice in cages enriched with tissues. Water and standard pellet food are provided ad libitum. All mice are monitored daily for daily discomfort. Animals displaying overt signs of discomfort (lack of activity or feeding behavior) are euthanized immediately by cervical dislocation.

### Method Details

#### Cytoplasmic injection and nuclear transplantation to *Xenopus* oocyte

This section was adapted from (Jullien, 2015).

##### Oocytes preparation

Ovaries are removed from a female *Xenopus laevis* terminally anesthetised by subcutaneous injection of 400μl of MS222 (Ethyl 3-aminobenzoate methanesulfonate, 0.6g/ml in H_2_0) into the dorsal lymph sac. The ovaries are then placed in a petri dish containing 1X MBS (MBS (Barth-HEPES Saline) 10X stock: 88mM NaCl, 1mM KCl, 2,4mM NaHCO3, 0.82mM MgSO4.7H2O, 0.33mM Ca(NO3)2.4H2O, 0.41mM Cacl2.6H2O, 10mM HEPES. Add ∼3 mL of 10N NaOH to obtain a pH of 7.4 to 7.6), and separated into small clumps (containing roughly 50-100 oocytes) using forceps. Three to five ml of such clumps are transferred to a 50 mL falcon tube that is then filled to 12.5 mL with 1X MBS. 6.5 units of liberase are then added to the solution and the oocytes are incubated 2h20 min at room temperature (22°C) with gentle agitation on a rocker (Liberase is prepared by resuspending 100 mg of lyophilized enzymes (Liberase TM Research / Roche 05 401 127 001) with 10 mL sterile H2O (giving a final concentration of 26 U/ml). Let the solution stand for 30 min on ice to rehydrate. Gently swirl the vial every few minutes to ensure that the enzyme is completely dissolved. Store aliquots of 250 μL (6.5U) at -80°C). Defolliculated oocytes are then washed 4 times in 50 mL of 1X MBS and transferred to a Petri dish. Individual oocytes are then manually sorted under a standard dissecting microscope using a Pasteur pipette and stored over night at 16-18°C in 1X MBS supplemented with penicillin streptomycin (P/S) at a final concentration of 10 μg/ml.

##### mRNA/gRNAs /BrUTP injection to oocyte’s cytoplasm

The oocytes are injected, in the cytoplasm, either one day prior (mRNAs) or two hours after (BrUTP, gRNAs) nuclear transfer. Using a Drummond injector (Drummond Nanoject, Drummond Scientific Company, USA), the required amount of mRNAs or gRNAs (9nl at 1mg/ml), or BrUTP (Sigma: B7166, 4.6nl at 100mM), is injected in the cytoplasm from the vegetal pole of the recipient oocyte. Injected oocytes are cultured in 1X MBS, P/S, 0.2%BSA at 18°C.

##### Donor cell preparation

Adherent cells such as mouse embryonic fibroblasts (MEF) or embryonic stem cells (ES) are collected by trypsinization according to standard tissue culture procedure. Trypsin treatment is stopped by addition of cell culture medium supplemented with serum. The cells are then washed twice in 1X PBS (137 mM NaCl; 2.7 mM KCl; 10 mM Na_2_HPO_4_; 2 mM KH_2_PO_4_; pH adjusted to 7.4 with HCl) by centrifugation for 3 min at 500 g. Prior to proceeding to permeabilization, single cells are collected by filtering the cell suspension through a 50 μm filters (Celltrics Partec 04-0042-2317). Cells are then counted on a hemacytometer and diluted to 1 million cells per ml. One million cells (in one ml of 1X PBS) are transferred to an eppendorf tube and centrifugated 2min at 2000 g at RT. The cell pellet is then resuspended in 1ml of SuNaSP (250mM Sucrose, 75mM NaCl, 0.5mM Spermidine, 0.15mM Spermine). The cells are centrifugated again and resuspended in 200 μL of SuNaSP. 25 μL of Streptolysin O (SLO, 500 units) is then added to the suspension and the cells are incubated 5 min at 37°C in a water bath (25000 units of Streptolysin O are resuspended in 1.25 mL PBS 0.01%BSA. 62.5 μL of dTT 100mM is then added to the solution and incubated 2 hr at 37°C. Aliquots of 25 μL are stored at -80°C). After incubation cells are placed on ice while the efficiency of permeabilization is checked. For that purpose 2 μL of the permeabilized cell suspension is mixed with 8 μL of 0.4% trypan blue solution (0.4 gr trypan blue diluted in 100 mL of PBS 1X). Cells in trypan blue are visualized on a hematocytometer. Permeabilization is successful when more than 90% of the cells show a nucleus stained blue and blebs are apparent on the cell plasma membrane. When permeabilization is confirmed to be satisfactory by the trypan blue test, SLO is inactivated by adding 1 mL of SuNaSP 3%BSA to the 225 μL of permeabilized cells. Following SLO inactivation, cells are centrifugated 2min at 2000 g at RT. The permeabilized cells pellet is then resuspended in 55 μL of SuNaSP-BSA to give a final concentration of 250 nuclei/13.8nl of solution. The permeabilized cells (“nuclei”) are then used for transplantation without further manipulation.

##### Transplantation procedure

Transcriptional reprogramming happens only when the nuclei are transplanted into the recipient oocyte own nucleus, the germinal vesicle (GV). The protocol below explains how to transplant the nuclei and subsequently check for successful GV targeting. First prepare the injection needle: glass capillaries (7” Drummond #3-000-203-G/XL glass capillaries) are pulled in a Flaming/Brown micropipette puller (Sutter instrument model P87 or p-7) using the following parameters: heat: set at the temperature established by a ramp test, pull: 100, velocity: 100, time: 10. The needle tip then needs to be cut to give a relatively large opening (∼50 μm) to be able to suck the donor nuclei suspension into the needle This can be done simply by using a dissecting scissor positioned at a 120 degree angle to the needle. This will give a sharp enough needle tip that will penetrate the oocyte without damaging it. It then is helpful to mark the needle with a marker pen at a distance of about 300 μm from the tip.The needle prepared in this way is filled with about 500 nL of the nuclei suspension containing 5pg/13.8nl of a PCS2+-membrane GFP plasmid. To inject the nuclei, the oocyte is positioned toward the needle with forceps. The needle has to contact the middle of the pigmented (animal) pole of the oocyte and be orientated parallel to the animal/vegetal pole axis. Once positioned, the needle is inserted in the oocyte at a depth of about 250 μm (using the pen mark as a reference) and the nuclei can then be injected (13.8nl containing 250 nuclei and 5pg of membrane GFP plasmid). GV targeting success rate has to be checked for each injection needle used since slight differences in the size of the oocyte and the sharpness of the needle affect the injection depth required. This is done by isolating the GV from the oocyte in MBS 1X and verifying the presence of nuclei in the GV. If the nuclei are not transplanted to the GV, the targeting test is repeated varying the injection depth, using the marker pen reference as a guide. The nuclei suspension is kept on ice and the needle is refilled with nuclei from the stock solution every 20-30 oocytes injected. It is important to note that very often the nuclei suspension, when sucked into the needle, does not remain homogeneous. Indeed the nuclei tend to become more concentrated at the oil/nuclei solution interface. For that reason it is crucial to refill the needle frequently and to then immediately transplant the nuclei to oocyte. This operating mode ensures that the quantity of nuclei delivered to the GV is constant. After transplantation, the oocytes are cultured in 1X MBS supplemented with antibiotics (penicillin/streptomycin, gentamycin) and 0.2% BSA. The oocytes are kept at temperature of 18°C and the culture medium is replaced every day. 24 hr after nuclear transplantation, oocytes with successful GV targeting are sorted based on the presence of GFP fluorescence at the oocyte plasma membrane (The membrane GFP RNA is produced only if the membrane GFP plasmid, and co-injected nuclei, are delivered to the oocyte GV). Transcriptional reprogramming is typically assessed between 0 and 3 days after transplantation from GFP positive oocytes.

#### Analysis of transplanted *Xenopus* oocytes

##### Gene expression from transplanted nuclei

###### RNAs extraction

Pool of 8 oocytes are collected in eppendorf tubes and can be stored at -80°C or directly processed for mRNA extraction. For each experimental condition, 2 samples of 8 oocytes are processed. RNAs are extracted using RNAeasy mini kit (QIAGEN 74106), according to the manufacturer instruction, with ON-column DNase treatment and a 50 μL final elution volume

##### RNA immunoprecipitation

###### Beads saturation

Anti BrdU-agarose beads (Santa Cruz Biotechnology SC 32323, 25 μL /IP) are washed twice by adding buffer I (0.5X SSPE (0.5mM EDTA and 74.5mM NaCl in 5mM phosphate buffer (pH 7.4) with 0.05% Tween20 and 0.1% polyvinylpyrrolidone [PVP, SIGMA P5288])and spinning 2’ at 2rcf. Beads are then blocked overnight on a rotating wheel at 4°C (up to 400 μL beads in 900 Buffer I supplemented with 100 μL of 10mg/ml RNase free BSA).

##### RNA immunoprecipitation

For each IP, 80 μL of RNA (40 μL from each samples) are added to 25 μL of saturated beads in 500 μL 0.5X SSPE with 0.05% tween 20 and 6.25 μL of RNase inhibitor (Superase, Ambion AM2696) and placed on a rotating wheel for 4h at 4 degree. 20 μL of RNAs is kept as an input fraction for RT-qPCR analysis.

##### Beads washes

Washes are with 600 μL of buffer for 10 min on rotating wheel at 4°C. Between washes samples are spun 2’ at 2rcf. First wash is with low salt buffer (0.2X SSPE with 0.05% Tween20), followed by two washes with high salt buffer (0.5X SSPEwith0.05%Tween20 and 150mMNaCl). The last wash is with TET (10mMTris, 1mMEDTA, pH 8, and 0.05% Tween20. After the last wash, aspirate most of the liquid so that the beads are dry.

##### Elution

RNAs are eluted by adding 100 μL elution buffer (300mMNaCl, 5mMTris, pH 7.5, 1mMEDTA, 0.1% SDS, 20mMdithiothreitol) to the beads and incubate 5’ at RT. The beads are then centrifuged 2’ at 2rcf, and the supernatant containing the eluted RNAs collected. The procedure is repeated four times to collect a total of 400ul of eluted RNAs Eluted RNA are phenol/chloroform (1:1 Mix PH8) extracted, and then precipitated by adding 1ml EtOH 100%,40 μL Na acetate 3M, 1 μg of glycogen and incubating 20min on dry ice. RNAs are then pelleted by centrifugation 20min at 12000 rcf. Supernatant is discarded and the pellet is then washed with EtOH 70%, centrifuged 5min at 12000 rcf. The supernatant is removed, the pellets is dried for 5min at RT, and then resuspended in 12 μL H_2_0.

##### RT-qPCR

Annealing of oligodT primers to the RNAs is performed by incubating at 65°C for 5min 12 μL of RNA mixed with 0.5 μL of 100 μM oligodT(15) and 0.5 μL of 10mM dNTPs followed by an incubation 5min on ice. cDNAs synthesis was performed by adding 4 μL of 5X second strand buffer, 1 μL 0.1M dTT, 1 μL H_2_O, 0.5 μL RNase inhibitor, and 0.5 μL superscriptIII reverse transcriptase (InVitrogen, 18080093) to the RNA solution and incubating at 50°C for one hour. The reverse transcription is stopped by adding 80 μL of H_2_O to the reaction and incubating 10 min at 70°C. For qPCR, 5 μL of cDNAs were mixed with 12.5 μL SyberGreen (SIGMA, S9194), 7.22 μL H_2_O, 0.14 μL each of Forward and Reverse primers (10 μM, see Table S8). The qPCR reaction was then performed on StepOnePlus System (applied biosystem) using the standard detection mode option.

##### RNA-seq

To eliminate any trace of solvent after phenol extraction, RNA are further purified using ARCTURUS PicoPure RNA Isolation Kit (Thermo Fisher, KIT0204) following the manufacturer instructions. 2 μL of purified RNAs (from a 10 μL volume of purified RNAs) are then used to generate RNA-seq library using Ovation Single Cell RNA-Seq System (Nugen), following the two steps PCR option of the manufacturer protocol.

##### WB analysis of histone modifications in transplanted nuclei

The GV containing transplanted nuclei are dissected out of the oocyte in GV isolation buffer (20mM Tris-HCl, pH7.5, 0.5mM MgSO4, 140mM KCl). Within 1-2 min following dissection, the isolated GVs are transferred on ice to an eppendorf tube containing non-denaturing wash buffer (150mM Tris-HC,l pH7.5, 50mM NaCl, 150mM KCl) supplemented with proteases inhibitors (1000X stock solution: AEBSF, 104 mM; Aprotinin, 80 μM, Bestatin, 4 mM, E-64, 1.4 mM, Leupeptin, 2 mM, Pepstatin A, 1.5mM). The operation is repeated until about 30 GVs are collected (corresponding to about 7500 transplanted nuclei). The GV containing the transplanted nuclei are then disrupted by brief vortexing (5 s), and centrifugated for 3 min at 12000 g. The supernatant is discarded and the pellets containing the nuclei and GV membrane is washed twice in 1ml of non denaturing washing buffer by vortexing 5 s and centrifugating for 3 min at 12000 g. The obtained pellets correspond mostly to transplanted chromatin components since the washing process gets rid of most of the GV proteins that are not tightly bound to the transplanted nuclei. The pellets can be frozen or directly processed for SDS-PAGE electrophoresis and WB analysis following a standard protocol. Quantitative immunoblotting was carried out on a LI-COR Odyssey CCD (charge-coupled device) scanner according to the manufacturer’s instructions (LI-COR Biosciences).

##### ChIP analysis

###### Chromatin preparation

Oocytes are washed in MBS 1X without BSA prior to fixation. Batch of oocytes (typically 56-84 oocytes per experimental conditions) are transferred to a 15 mL falcon tube. Eight ml of 1X MBS containing 1% formaldehyde are added to the oocytes and the samples are fixed for 10 min at room temperature with gentle agitation. The fixation is stopped by the addition of 4ml of MBS 1X with 300mM glycine. The oocytes are then washed three times in 10ml MBS 1X. Groups of 7 fixed oocytes are transferred to eppendorf tubes in which MBS is replaced by 280 μl of homogenization buffer (50mM HEPES, pH7.8, 140mM NaCl, 1mM EDTA, 1% Triton X-100, 0.1% Na-deoxycholate) supplemented with 0.3% Sodium Dodecyl Sulfate (SDS), proteases inhibitors, and 1mM DTT. Oocytes are thoroughly disrupted by pipetting up and down using a micropipette. Sonication is carried out in two cycles of 7 min on a Sonicator equipped with an ultrasonic bath (with 30 s on/off cycles, higher power). After sonication, the SDS in the sample is diluted by adding 600 μl of homogenization buffer supplemented with proteases inhibitors and 1mM DTT. The sonicated chromatin is then centrifugated at 12000 g for 10 min at 4 degree. The supernatant (including the lipid layer) is then transferred to a new tube. This chromatin can then be used in a standard ChIP protocol.

##### Chromatin immunoprecipitation

10% of the chromatin is taken as input and mixed with an equal volume of 2 × Stop buffer (40 mM Tris-HCl at pH 8.0, 10 mM EDTA, 1% SDS) and 0.3 μg/μL proteinase K (final concentration). The inputs are incubated overnight at 65°C. The rest of the sonicated solution is mixed with antibody and incubated overnight at 4°C on a rotating wheel. The antibodies used are: 1 μL of rabbit anti-HA antibody (ab9110, Abcam) or rabbit anti H2AK119Ub1 antibody (cell signaling, 8240). After antibody reaction, 20 μL of dynabeads protein G (Invitrogen) is added and is rotated for another 6 hr at 4°C. The bead antibody conjugates are sequentially washed with buffer I, buffer II (50 mM HEPES at pH 7.8, 500 mM NaCl, 1 mM EDTA, 1% Triton X-100, 0.1% sodium deoxycholate, 1 mM DTT, protease inhibitors), buffer III (20 mM Tris-HCl at pH 8.0, 1 mM EDTA, 250 mM LiCl, 0.5% NP40, 0.5% sodium deoxycholate, 1 mM DTT, protease inhibitors), and TE containing 1 mM DTT and protease inhibitors for 30 min at 4°C with rotation. After the last wash, 150 μL of 1 × Stop buffer with 0.3 μg/μL proteinase K is added to the beads, and cross-links are reversed by incubation overnight at 65°C. DNA from inputs and immunoprecipitates are then purified by phenol/extraction and ethanol precipitation and resuspended in a final volume of 50 μL of H_2_O.

##### ChIP qPCR and ChIP-seq

DNA is processed for qPCR analysis as described in the RT-qPCR section. Library for ChIP-seq were generated using TruSeq DNA Sample Prep Kit (illumina; FC-121-2001) according to the manufacturer instructionsS8

#### Plasmid constructs and in vitro RNA synthesis

Human Kdm4D, mouse KDM6B (aa1025-1642), human USP21, and *Xenopus laevis* ring1B I49A were cloned using p-Entry cloning system (Invitrogen, K2400-20 and 11791-020) into a pCS2+ destination plasmid with three C-terminal HA-tags and an N7-NLS-tag (Cutler et al., 2000). mRNAs were synthesized in vitro using MEGAscript SP6 Kit (Ambion, AM1330M) following the manufacturer’s instructions. Templates for in vitro synthesis of guide RNAs were produced by mixing 20 μL 5X HF buffer (M0530, New England biolabs), 10 μL dNTPs (2.5mM), 10 μL guide specific Forward primer (2 μM, see Table S8), 10 μL common guide Reverse primer (2 μM, see Table S8), 1 μL Phusion High Fidelity DNA Polymerase (M0530, New England biolabs) and running a PCR reaction with the following parameters: 30” initial denaturation at 98°C, then 35 repeat of 15” denaturation at 98°C, 30” annealing at 30°C, and 15” elongation at 72°C. The PCR reaction products were purified using QIAquick PCR Purification Kit (cat#28104, QIAGEN) according to the manufacturer instructions and using a 50 μL elution volume. 8 μL of the purified PCR product (∼0.5 μg of DNA) was then used in an in vitro RNA synthesis reaction with MEGAscript T7 Kit (Ambion, AM1334) following the manufacturer’s instructions (20 μL total reaction volume).

#### Mouse nuclear transfer

Oocytes were prepared by superovulating eight-week old C57/CBAF1 mice. Superovulation was induced by injecting pregnant mare serum gonadotropin (PMSG, Intervet, 5 UI) and human chorionic gonadotropin (hCG, Intervet, 5 UI) at intervals of 48 hr. Oocytes were collected from oviducts 14 hphCG (hours after injection of hCG) and washed in M2 medium containing 300 IU/ml hyaluronidase (Sigma, H4272) to remove cumulus cells. Subsequently, they were incubated in M2 (Sigma, M7167) containing 5 μg/ml cytochalasin B (SIGMA C6762) and placed in the same medium in a chamber on the stage of an inverted microscope (Olympus IX70) equipped with micromanipulators (Narishige MO-188). The chromatin spindle (visualized under differential interference contrast) was aspirated into the pipette and removed. Donor nucleus, from ES cell synchronized in metaphase by 2 hr culture in medium containing 0.05 μg/ml of demecolcine (SIGMA D7385), were then isolated by gentle aspiration of the cell in and out of the injection pipette (inner diameter 7–8 μm). Microinjected into the cytoplasm of the enucleated oocytes was then performed by Piezo-assisted technique. The nuclear transfer embryos were activated by incubation for 3 hr in Ca^2+^-free medium containing 10 mM Sr2+. Embryos with visible nuclei were considered as activated, were transferred into fresh M16 medium (Sigma, M7292) and cultured at 37°C in a humidified atmosphere containing 5% CO2. For microinjection embryos were placed in M2, in a chamber on the stage of a Nikon inverted microscope equipped with Narishige micromanipulators and an Eppendorf microinjector. Embryos were microinjected in the cytoplasm with 1-2 pl of 1μg/μl USP21 mRNA, yielding an approximate dilution of 1/2000. Microinjections were performed at 4-5 hr post-activation. Incubation was then carried out in M16 culture medium at 37°C, 5% CO2 for further development.

#### Mouse embryos immunostaining

Embryos were fixed by placing them in 2% paraformaldehyde (PFA, EMS) in PBS for 10 min at room temperature and then permeabilized for 15 min at room temperature using 0.5% Triton X-100 in PBS (Sigma-Aldrich). The embryos were incubated in 2% BSA-PBS for 1 hr (to block unspecific binding sites) and processed for *in toto* immunolabelling overnight at 4°C with anti-ubiquityl-histone H2A (Lys119) antibody (Cell Signaling, #8240S) diluted 1/200 in 2% BSA-PBS. After being washed two times 15 min with PBS, the embryos were incubated for 1 hr at room temperature with an anti-rabbit AlexaCy3-conjugated secondary antibody (Jackson ImmunoResearch Laboratories Inc., USA), diluted (1/200) in 2% BSA-PBS. DNA counterstaining was performed for 15 min at 37°C using 5μg/ml DAPI (Invitrogen) in PBS. The embryos were washed using PBS and gently mounted on slides using a large amount of Citifluor antifading agent (AF1 BioValley) to preserve the 3D structure of the nuclei.

#### Filtering and mapping of sequencing data

RNA-seq and ChIP-seq libraries were sequenced on an Illumina HiSeq 2000 instrument in single read mode at 40 or 50 base length. Fastq files were filtered for low quality reads (< Q20) using sickle (Joshi and Fass, 2011) and low quality bases were trimmed from the ends of the reads (< Q20). Adapters were removed using cutadapt 1.0 (Martin, 2011). For samples corresponding to mouse nuclei transplanted to *Xenopus* oocytes, sequencing reads corresponding to mouse were obtained by mapping to a background genome containing Mouse mm9 and *Xenopus laevis* 6.1 (Bowes et al., 2008). The sequencing reads were mapped to this background genome using bwa 0.6.2 (Li and Durbin, 2009). Samtools 0.1.8 was used to generate sorted bam files from the output of bwa (Li et al., 2009). Reads that mapped exclusively to a single genome were extracted with a further quality filter of mapping quality > 13 being applied using bamtools 2.1.1 (Barnett et al., 2011). This resulted in BAM files representing the reads that exclusively map to each genome and were used for further analysis. Single genome RNA-Seq samples were mapped with TopHat version 2.0.6 (Trapnell et al., 2009) along with a junction file based on the *Mus musculus* UCSC mm9 genome with RefSeq annotation. Reads were generated for each gene by summing the number of mapped reads overlapping its exons. These were converted to RPKMs by normalizing by the total read count for each sample and corresponding transcript lengths.

### Quantification and Statistical Analysis

#### Description of biological replicates

##### RNA-seq

For *Xenopus* oocytes nuclear transfer experiments, two biological replicates (using different batch of oocytes and different donor cell preparations) were used in each cases. RNA-seq library corresponding to one replicate originated from RNAs purified from 32 *Xenopus* oocytes each transplanted with ∼250 mouse nuclei. For nuclear transfer to mouse oocyte, three biological replicates were used using different batch of oocytes and different donor cell preparations. In this case, RNA-seq library corresponding to one replicate originated from RNAs purified from 7 to 10 two-cell stage cloned embryos. RT-qPCR: three to four biological replicates were used (using different batch of oocytes and two different donor cell preparations). Within one replicate 3 samples with 8 NT-oocytes are used for each experimental conditions. ChIP-Seq: two biological replicates (using different batch of oocytes and different donor cell preparations) were used in each case. ChIP-seq library corresponding to one replicate originated from gDNAs purified from 80 *Xenopus* oocytes each transplanted with ∼250 mouse nuclei. ChIP-qPCR: Four biological replicates were used (using four different batch of oocytes and two different donor cell preparations). Within one replicate 3 to 4 samples with 10 NT-oocytes are used for each experimental conditions.

#### Gene Expression Analysis

Counts were generated by overlapping mapped reads with exonic positions defined by the UCSC RefSeq gtf file for mm9. RPKM values were then generated through normalization to total read counts and transcript length. Where replicates were available for both sample types in a pairwise comparison, transcripts were kept in the analysis if they had at least one count per million (CPM) in either all samples of one type or all samples of the other type. Differentially expressed genes were identified in R (version 3.2.4) with EdgeR (Robinson et al., 2010) by calculating the log2 fold change (logFC) and the false discovery rate (FDR). A threshold of FDR < 0.05 was then used to select differentially expressed genes. Where replicates were not available on both sides of a comparison, transcripts were kept if they had RPKM > 1 in all samples of either type. Differential expression was then defined by an absolute value greater than one of the log fold change of the mean expression for each sample type in the pairwise comparison. For MA-plot, the log2 FC in expression of transcripts between MEF-NT to ES-NT was plotted against the average donor cell gene expression (log2(RPKM+1). Box-plots show distribution of mean gene expression levels of the different sets of transcripts. The middle line in the box indicates the median, the box edges indicate the 25th/75th percentiles, the whiskers indicate the min and max. All plots are produced using the boxplot command in R. Differences in gene expression levels between pairwise sets of genes were tested using Mann-Whitney test (equivalent to Wilcoxon rank sum test). For RT-qPCR analysis the indicated genes were quantified using a standard curve of mouse genomic DNA. The data was then visualized as mean value in a column bar graph with the standard error of the mean as error bar. When indicated, significance was calculated using Student t test (★, P value < 0.05). Heatmaps: For our newly generated expression data, the RPKMs used for heatmaps are averaged over the two replicates. For Figure 1C, a mean is taken for each condition. Samples taken before NT and after NT are then internally normalized by the average value before NT and after NT, respectively, and a log is taken. Finally, each sample is z-scored over all selected genes and the genes are ordered by fold change from ES to MEF after NT. Expression data for cells before NT were taken from (Reddington et al., 2013) and (Bulut-Karslioglu et al., 2014). In Figure 4F, expression values are z-scored and then split into groups using k-means clustering. In Figure 5E, the values indicated are log2(1+RPKM) and they are reordered by unsupervised hierarchical clustering. Heatmaps were plotted using the heatmap.2 function in R from the package gplots (Warnes et al., 2016) using z-scored expression values. For Figure 1C and Figure 4F, no further clustering or reordering was performed by the package. For Figure 5E, rows were reorderded by the default unsupervised hierarchical clustering (parameter Rowv = TRUE).

#### ChIP Analysis

For ChIP-Seq analysis, reads were counted in each 200bp bin for 10kb around the TSS of every genes. These counts were then summed over all genes in each gene set of interest and normalized to the number of genes in the set, as well as the total read count for the sample. For each IP the corresponding values for the paired input were subtracted. For ChIP-qPCR, the indicated genes were quantified using a standard curve mouse genomic DNA. For normalization, the values of the IP were represented as percent of the Input values. The data was then visualized as mean value in a column bar graph with the standard error of the mean as error bar.). When indicated, significance was calculated using Mann-Whitney test (equivalent to Wilcoxon rank sum test; ★, P value < 0.05)

#### GO and KEGG pathway enrichment

Analysis of enrichment on MEF resistant and ES resistant genes were carried out with DAVID using all mm9 genes as a background list.

#### Mouse embryo image quantitation

The embryos were observed using a Carl Zeiss (Germany) AxioObserver Zl fluorescence microscope equipped with the ApoTome slider (MIMA2 Platform, INRA). Z series acquisition feature every 0.4 μm with a 63x Plan-Neofluar oil objective (NA 1.3) and the same settings (acquisition time/laser power/gain/offset) for all samples. Raw images are available on the Mendeley repository: http://dx.doi.org/10.17632/jzjspk58nv.1, http://dx.doi.org/10.17632/2nm2rvcx2y.1, http://dx.doi.org/10.17632/ckzm2npz6h.1. The staining levels were estimated by quantifying fluorescence signals as follows, using Image-J software (National Institute of Health, Bethesda, Maryland, USA): 1) the z-range was determined for each nucleus to evaluate its thickness and its radii a/b were measured on the larger section; 2) the volume of each nucleus that is ellipsoidal was calculated with the mathematical formula v = 4/3π^∗^ab^∗^(thickness/2); 3) a summing z-projection was realized; 3) the area of each nucleus was outlined manually and the mean fluorescence intensity as well as the nucleus area were measured; 4) the total fluorescence intensities for each nucleus were calculated according to the following mathematical formula: total fluorescence = mean fluorescence ^∗^ volume of the ellipsoid. For each experimental replicate the average fluorescence levels of the injected group were normalized against the control group, which was set at 1 arbitrary unit, and the replicates were then compared with a non-parametric test from Mann Whitney using the R stats package.

### Data and Software Availability

The accession number for the ChIP-seq and RNA-seq datasets (40 *Xenopus* oocyte NT RNA-seq, 8 mouse egg NT RNA-seq, and 4 ChiP-seq samples) reported in this paper is GEO: GSE87872 (https://www.ncbi.nlm.nih.gov/geo/query/acc.cgi?token=ehgpswyqddmvzqt&acc=GSE87872).

## Author Contributions

Conceptualization, J.J. and J.G.; Methodology, J.J., K.M., and V.P.; Formal Analysis, G.A.; Investigation – *Xenopus*, J.J. and M.V.; Investigation – Mouse, J.J., S.A.D., V.B., and N.B.; Writing – Original Draft, J.J.; Writing – Review & Editing, J.J., V.P., and J.G.; Resources, C.B., S.W., H.K., M.O., K.M., and V.S.; Data Curation, C.B.; Visualization, J.J. and G.A.; Supervision, J.J. and J.G.

## Supplemental Information

Document S1. Figures S1–S5

Table S1. Differential Gene Expression Analysis between MEF-NT and ES-NT, Related to Figure 1

Table S2. Gene Ontology Terms and KEGG Pathways Enriched in Resistant Genes, Related to Figure 1

Table S3. Differential Gene Expression Analysis between ES-NT and MEF-NT in 12 Chromatin Configurations, Related to Figure 3

Table S4. Digital Signature of MEF-NT-Resistant Gene Sensitivity to Chromatin Modifiers, Related to Figure 4

Table S5. Differential Gene Expression Analysis among Control, USP21, Ring1a&b KO, and Xlring1B Mutant MEF-NT, Related to Figure 5

Table S6. Differentially Expressed Genes among Control Fertilized, Control Clone, and USP21 Clone Two-Cell-Stage Mouse Embryos, Related to Figure 6

Table S7. Downregulated or Upregulated Genes in Control and USP21 Clone Embryos, Related to Figure 6

Table S8. qRT-PCR, ChIP-qPCR, and gRNA Primers Used in the Study, Related to Figures 1, 4, and 5

Document S2. Article plus Supplemental Information
