# Supplementary material for: Gene Resistance to Transcriptional Reprogramming following Nuclear Transfer Is Directly Mediated by Multiple Chromatin-Repressive Pathways
Source: Mol Cell. 2017 Mar 2;65(5):873–884.e8. doi: 10.1016/j.molcel.2017.01.030 (PMC5344684; doi:10.1016/j.molcel.2017.01.030)
Supplement: Document S1. Figures S1–S5 [file mmc1.pdf]

Molecular Cell, Volume *65*

Supplemental Information

Gene Resistance to Transcriptional Reprogramming following Nuclear Transfer Is Directly Mediated by Multiple Chromatin-Repressive Pathways

Jerome Jullien, Munender Vodnala, Vincent Pasque, Mami Oikawa, Kei Miyamoto, George Allen, Sarah Anne David, Vincent Brochard, Stan Wang, Charles Bradshaw, Haruhiko Koseki, Vittorio Sartorelli, Nathalie Beaujean, and John Gurdon

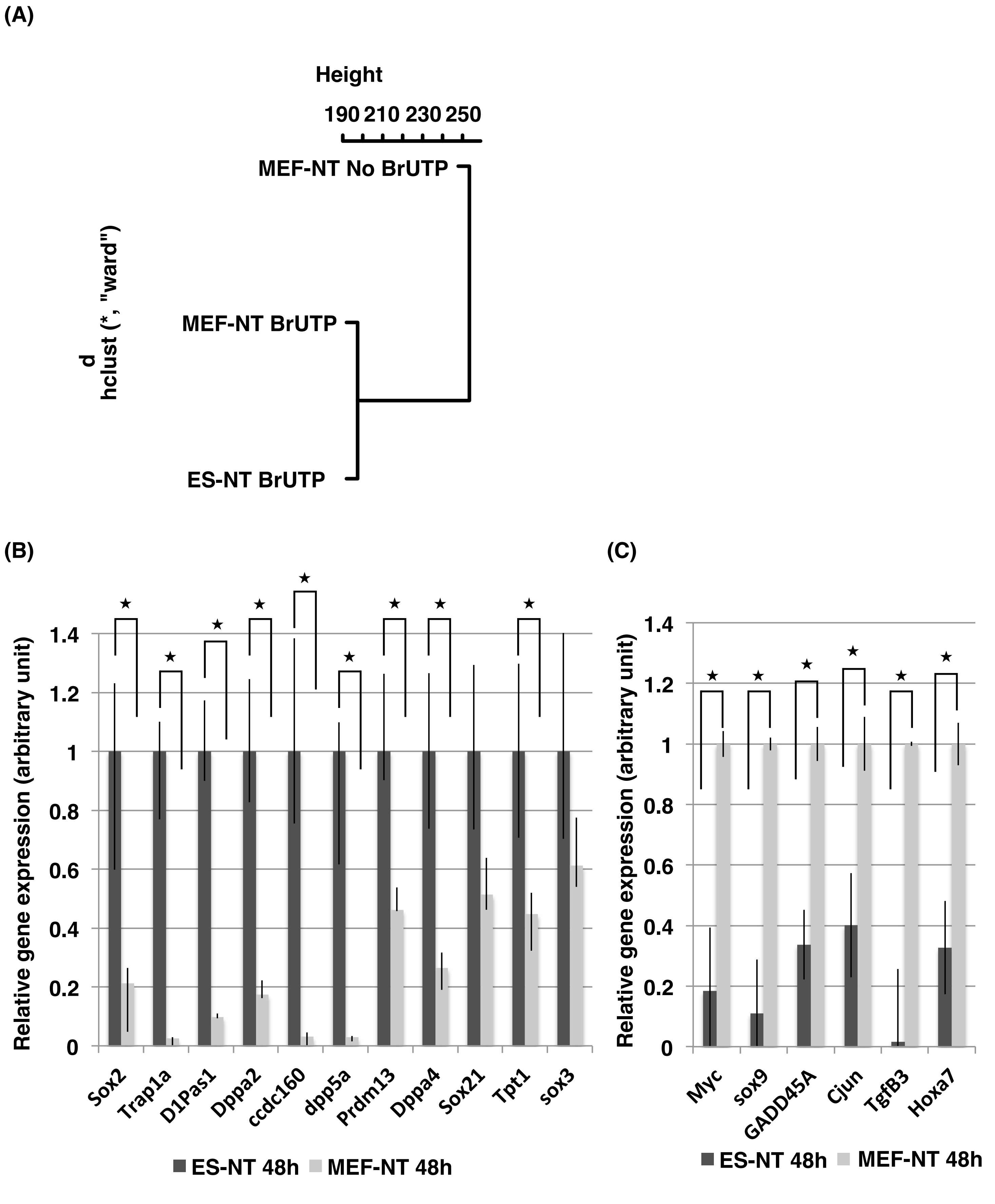

Supplemental Figure S1 related to Figure 1

(A)

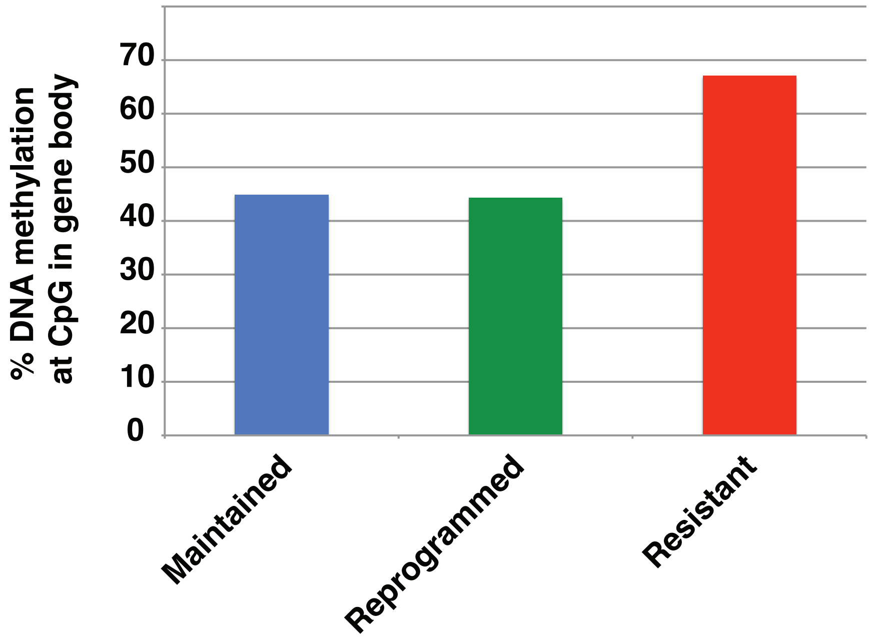

(B)

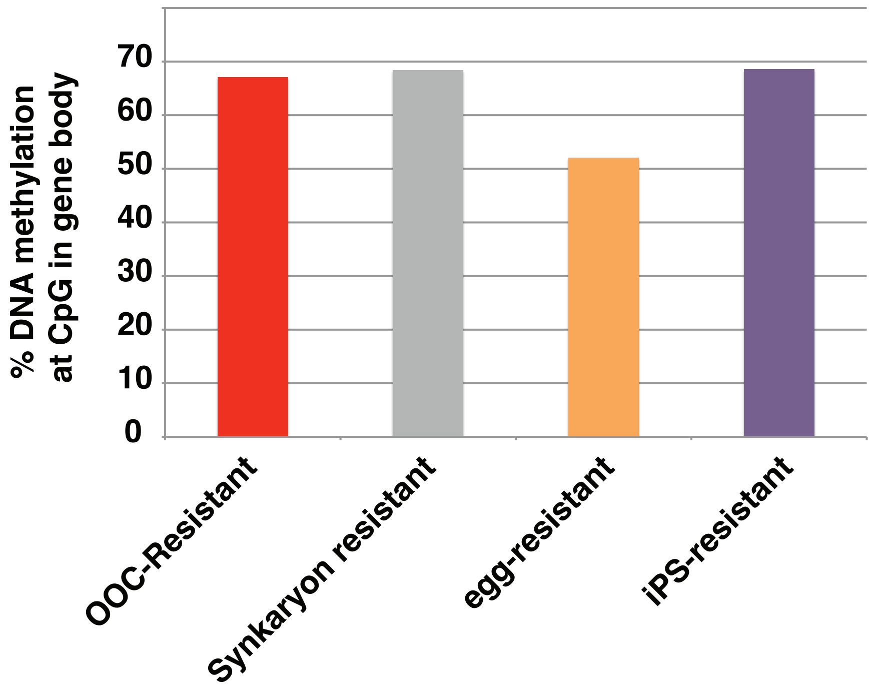

Supplemental Figure S2 related to Figure 2

(A)

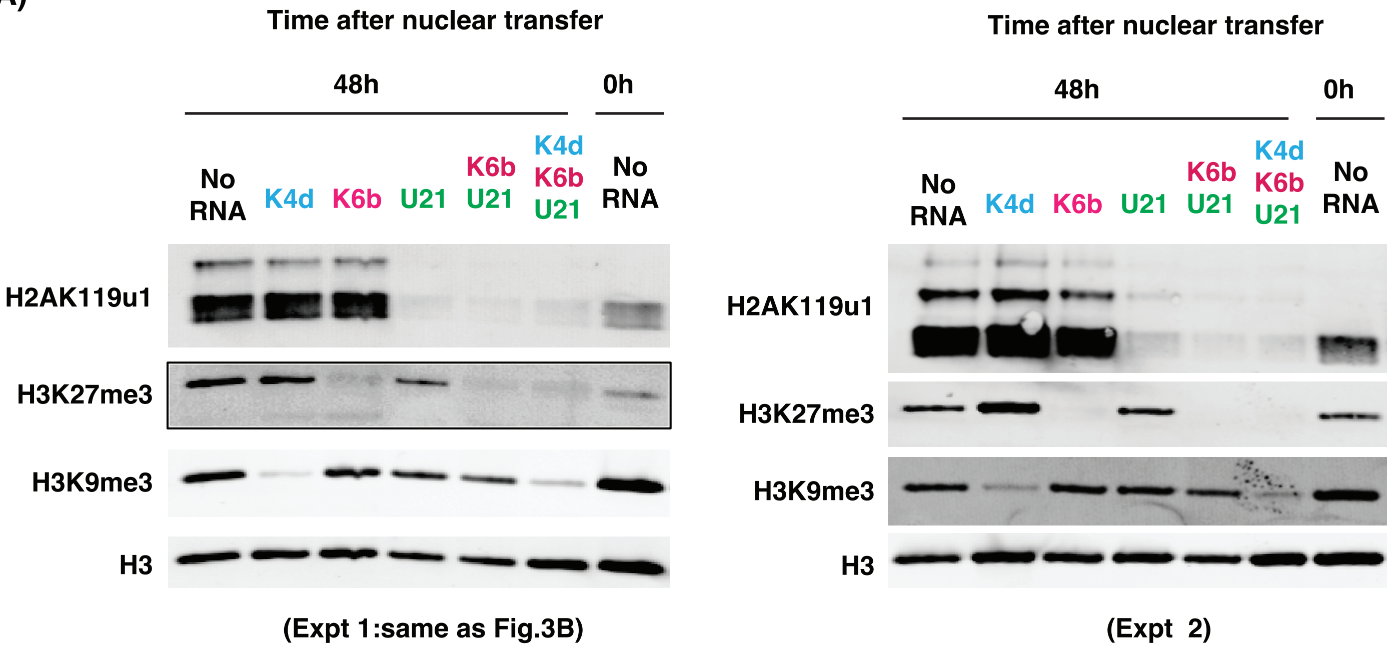

(B)

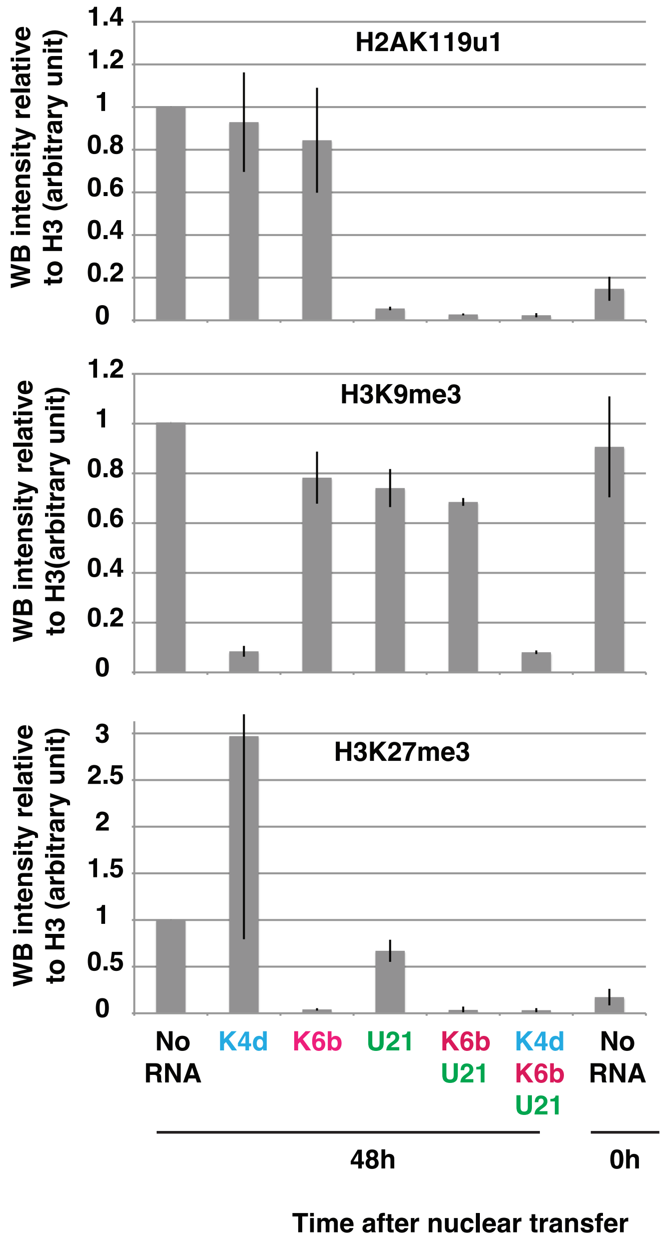

Supplemental Figure S3 related to Figure 3

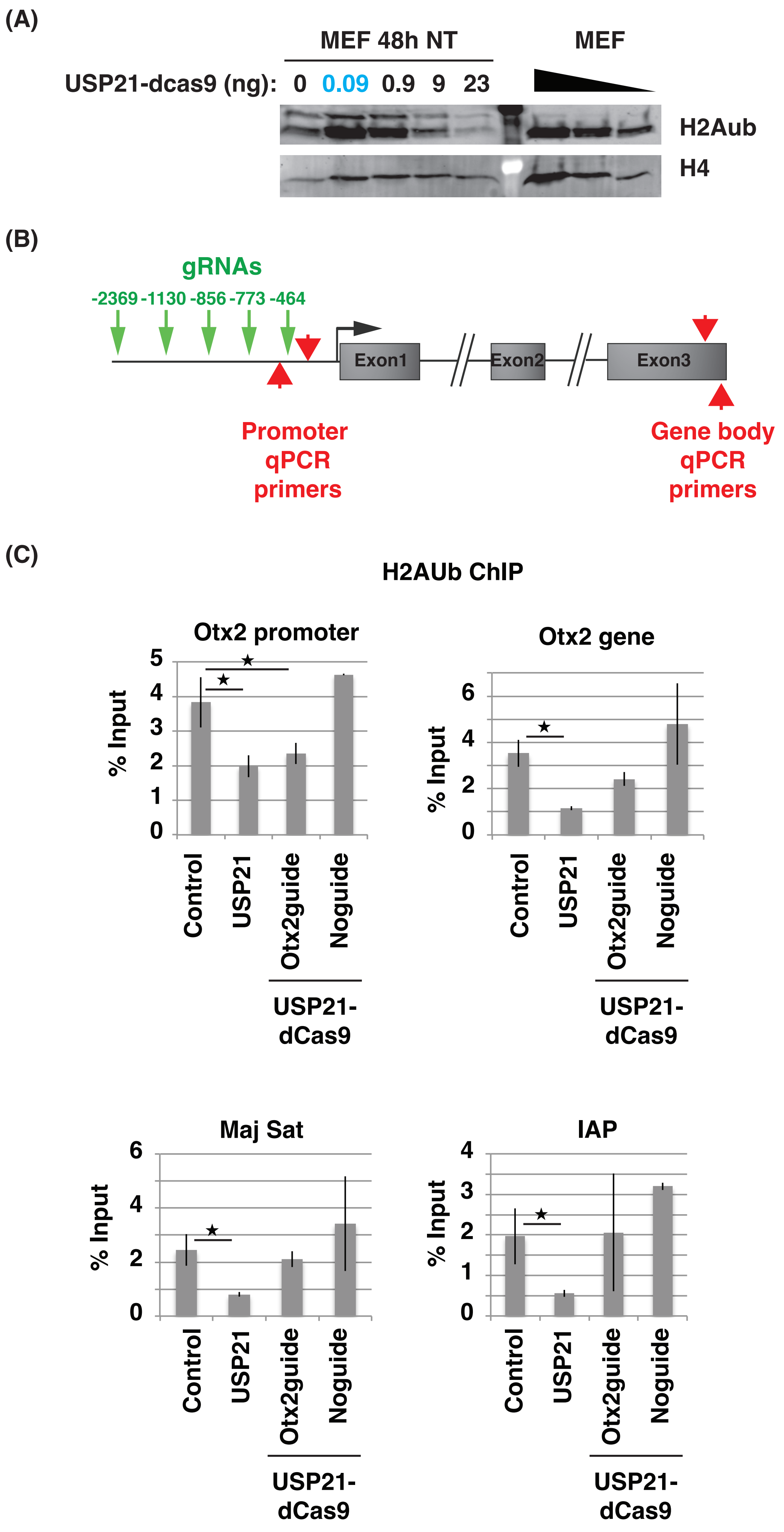

Supplemental Figure S4 related to Figure 5

(A)

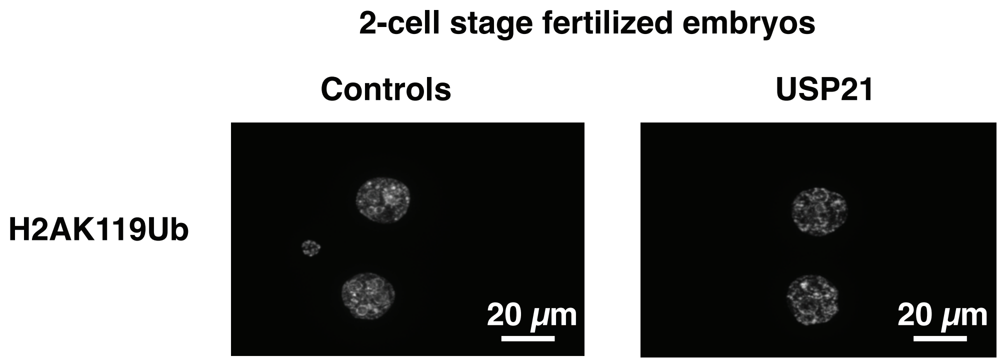

(B)

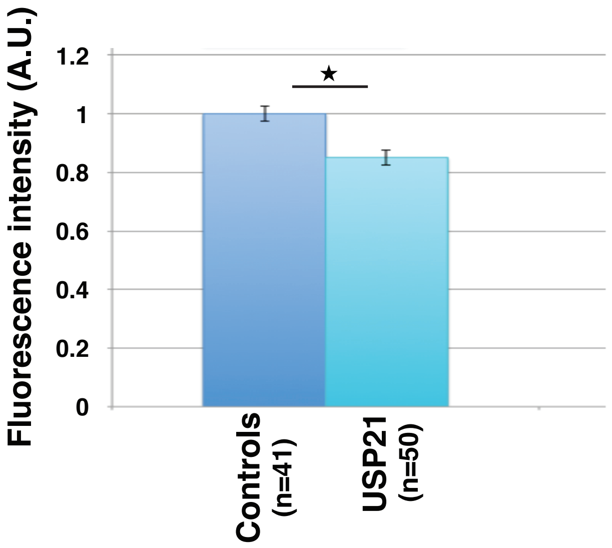

Supplemental Figure S5 related to Figure 6

SUPPLEMENTARY MATERIALS:

LEGENDS TO FIGURES S1-S5:

LEGENDS TO TABLES S1-S8:

Figure S1 related to Figure 1: *Identification of resistant genes by RNA-seq analysis of BrUTP labelled RNA 48h after nuclear transfer*

(A) hierarchical clustering of RNA-seq data obtained from samples obtained with (MEF-NT and ES-NT BrUTP)or without BrUTP labelling (MEF-NT No BrUTP) . **(B)(C)** RT-qPCR analysis of genes identified as resistant in MEF (B) or ES (C). Mean +/- sem from n=3 experiments. (★, P value< 0.05, student t-test).

Figure S2 related to Figure 2: *CpG methylation in the gene body of resistant genes*

(A) DNA methylation in the gene body of reprogrammed , maintained or resistant genes shown as percentage of CpG showing >95% methylation (data from (Reddington et al., 2013),). **(B)** DNA methylation in gene body of genes resistant in OOC-NT, mouse egg-NT, transcription factor induced reprogramming, and cell fusion shown as percentage of CpG showing >95% methylation (data from (Reddington et al., 2013),).

Figure S3 related to Figure 3: *Quantitation of histone modification removal from transplanted nuclei upon chromatin modifier expression*

(A) Western blot analysis of histone modifications on the chromatin of transplanted nuclei collected prior nuclear transfer (0h), or 48h after nuclear transfer to oocytes expressing various combinations of chromatin modifiers. The left and right panels show analysis corresponding to the two experiments used for the RNA-seq analysis. **(B)** Quantitation of modified histones signal in transplanted nuclei. Intensity of modified histones was averaged from the two experiments shown in B and normalised to average total histone H3 signal intensity obtained by fluorescence measurement (LI-COR detection system). Mean +/- sem from n=2 experiments .

Figure S4 related to Figure 5: *USP21-dCas9 mediated H2A deubiquitylation of Otx2*

(A) Titration of USP21-das9 mRNA injection. Oocytes were injected with 0 to 23 ng of USP21-dcas9 mRNA 24h prior to nucler transplantation. 48h after nuclear transfer the transplanted chromatin is recovered and analysed by western blot for H2AK119u1 level. Injection of 0.09 ng of USP21-dcas9 does not globally affect H2AK119u1 level in transplanted chromatin and this amount of mRNA was therefore used for subsequent targeting experiments. **(B)** Location of guide RNAs targeting the *Otx2* promoter (green arrows) as well as the primers used in ChIP-qPCR experiment (red arrows). **(C)** H2AK119u1 ChIP for *Otx2* promoter,

Otx2 gene body, and repeat elements of the genome (major satellite and IAP) following nuclear transfer to control oocytes, USP21 (9ng) injected oocytes, or USP21dcas9 injected (0.09ng) oocytes together with or without co-injection of *Otx2* guide RNAs mix. (★, P value< 0.05, Mann-Whitney test). Mean +/- sem from n=2 experiments.

Figure S5 related to Figure 6: *USP21 mRNA injection decreases* H2AK119u1 *level in mouse 2-cell embryos*

(A) H2AK119u1 in the nuclei of control or USP21 injected embryos. Embryos are injected at fertilization with mRNA encoding USP21 and subsequently fixed for immunolabelling at the 2-cell stage. **(B)** Quantitation of H2AK119u1 signal intensity in control and USP21 injected 2-cell stage embryos. Number of embryos quantitated shown in bracket. (★, P value< 0.001, Mann-Whitney test).

TABLES S1-S8:

Table S1 related to Figure 1: *Differential gene expression analysis between MEF-NT and ES-NT*

Table S2 related to Figure 1: *Full list of GO terms and KEEG pathways enriched in resistant genes*

Table S3 related to Figure 3: *Differential gene expression analysis between ES-NT and MEF-NT in 12 chromatin configurations*

Table S4 related to Figure 4: *Digital signature of MEF-NT resistant genes sensitivity to chromatin modifiers*

Table S5 related to Figure 5: *Differential gene expression analysis between control, USP21, ring1A&B KO, Xlring1B mutant MEF-NT*

Table S6 related to Figure 6: *Differentially expressed genes between control fertilised, control clone, USP21 clone two cell-stage mouse embryos.*

Table S7 related to Figure 6: *List of down- or up-regulated genes in control and USP21 cloned embryos.*

Table S8 related to Figure 1&4&5: *RT-qPCR, ChIP-qPCR and gRNA primers used in that study*
